# Amyloid-beta and tau pathologies act synergistically to induce novel disease stage-specific microglia subtypes

**DOI:** 10.1186/s13024-022-00589-x

**Published:** 2022-12-17

**Authors:** Dong Won Kim, Kevin J. Tu, Alice Wei, Ashley J. Lau, Anabel Gonzalez-Gil, Tianyu Cao, Kerstin Braunstein, Jonathan P. Ling, Juan C. Troncoso, Philip C. Wong, Seth Blackshaw, Ronald L. Schnaar, Tong Li

**Affiliations:** 1 Solomon H. Snyder Department of Neuroscience, Johns Hopkins University School of Medicine, Baltimore, MD 21205 USA; 2 Department of Pathology, Johns Hopkins University School of Medicine, Baltimore, MD 21205 USA; 3 Department of Pharmacology and Molecular Sciences, Johns Hopkins University School of Medicine, Baltimore, MD 21205 USA

**Keywords:** Amyloid-β (Aβ), Tau, Microglia, Alzheimer’s disease (AD), Sialic acid-binding immunoglobulin-type lectin (Siglec)

## Abstract

**Background:**

Amongst risk alleles associated with late-onset Alzheimer’s disease (AD), those that converged on the regulation of microglia activity have emerged as central to disease progression. Yet, how canonical amyloid-β (Aβ) and tau pathologies regulate microglia subtypes during the progression of AD remains poorly understood.

**Methods:**

We use single-cell RNA-sequencing to profile microglia subtypes from mice exhibiting both Aβ and tau pathologies across disease progression. We identify novel microglia subtypes that are induced in response to both Aβ and tau pathologies in a disease-stage-specific manner. To validate the observation in AD mouse models, we also generated a snRNA-Seq dataset from the human superior frontal gyrus (SFG) and entorhinal cortex (ERC) at different Braak stages.

**Results:**

We show that during early-stage disease, interferon signaling induces a subtype of microglia termed Early-stage AD-Associated Microglia (EADAM) in response to both Aβ and tau pathologies. During late-stage disease, a second microglia subtype termed Late-stage AD-Associated Microglia (LADAM) is detected. While similar microglia subtypes are observed in other models of neurodegenerative disease, the magnitude and composition of gene signatures found in EADAM and LADAM are distinct, suggesting the necessity of both Aβ and tau pathologies to elicit their emergence. Importantly, the pattern of EADAM- and LADAM-associated gene expression is observed in microglia from AD brains, during the early (Braak II)- or late (Braak VI/V)- stage of the disease, respectively. Furthermore, we show that several Siglec genes are selectively expressed in either EADAM or LADAM. *Siglecg* is expressed in white-matter-associated LADAM, and expression of *Siglec*-*10*, the human orthologue of *Siglecg,* is progressively elevated in an AD-stage-dependent manner but not shown in non-AD tauopathy.

**Conclusions:**

Using scRNA-Seq in mouse models bearing amyloid-β and/or tau pathologies, we identify novel microglia subtypes induced by the combination of Aβ and tau pathologies in a disease stage-specific manner. Our findings suggest that both Aβ and tau pathologies are required for the disease stage-specific induction of EADAM and LADAM. In addition, we revealed Siglecs as biomarkers of AD progression and potential therapeutic targets.

**Supplementary Information:**

The online version contains supplementary material available at 10.1186/s13024-022-00589-x.

## Background

Neuroinflammation is increasingly recognized as a key regulator of disease progression in neurodegenerative disorders [1, 2], including Alzheimer’s disease (AD), which is the most common cause of dementia [3]. Recent studies suggest that neuroinflammation serves as a mechanistic link between the development of amyloid-β (Aβ) plaques and tau neurofibrillary tangles, and the canonical pathologies of AD that are thought to drive synapse loss and neuronal death [3]. In addition to well-characterized AD susceptibility genes such as *APP, PSEN1 and PSEN2* (early-onset familial AD [4]), and *APOE* (late-onset AD [5, 6]), several additional risk alleles for late-onset AD were identified in genes regulating immunomodulation, including *TREM2 *[7, 8], phosphoinositide phospholipase Cγ2 (*PLCG2*) [9] and *CD33* [10, 11].

As the brain’s resident innate immune cells, microglia play multifunctional roles in brain health and the progression of neurodegenerative diseases such as AD [1, 2, 12]. Depending on the disease stage, microglia may protect against neurodegenerative proteinopathy and/or contribute to inflammatory damage [2, 13]. Microglia maintain brain health by clearing cellular debris, including Aβ plaques and tau aggregates [13]. Microglial activation correlates positively with cognition and gray matter volume in humans, indicating that microglia can be protective, at least during early-stage AD [14]. However, microglia also secrete proinflammatory cytokines and can directly contribute to tau pathology [14, 15] and its subsequent neurotoxicity [1, 2]. This dichotomous role of microglia in maintaining this balance between phagocytosis/clearance and pro-inflammatory mediator release is thought to be an essential and potentially targetable determinant of AD progression [16].

Recent studies have identified subtypes of microglia that display a dynamic range of responses and functions [17], emphasizing the ability of microglia to serve a variety of important physiological roles [12]. Transcriptomic analyses of bulk tissues revealed disease-associated changes in microglia associated with AD [18, 19]. Subsequent single-cell RNA-Sequencing (scRNA-Seq) approaches, however, were necessary to identify disease context-dependent microglial subtypes. scRNA-Seq has shed light on the spatial and developmental heterogeneity of microglia and provides a high-resolution view of the transcriptional landscape of microglia subtypes during development and disease progression [17, 20–23]. Since AD is a chronic disease with decades-long prodromal stages, understanding the disease stage-specific impacts of microglia subtypes is necessary to clarify the dual nature of microglia activation. The identification of disease-associated microglia (DAM), a unique TREM2-dependent subtype that expresses CD11c and is localized near Aβ plaques in an amyloidosis mouse model [20], supports this notion. Despite these advances, a critical unresolved question is whether disease stage-specific microglia subtypes exist that are activated in response to both Aβ and tau pathologies. These microglia subtypes would represent novel therapeutic targets for modifying disease progression.

Here, we used an AD mouse model (*Tau4RΔK-AP* mice), in which wild-type tau is converted into tau aggregates to drive neuron loss in a neuritic plaque-dependent manner [24]. This animal model serves as an excellent mouse model to profile microglia subtypes across different stages of AD-like disease progression. To assess the requirement for both Aβ and tau pathologies to induce disease stage-specific microglia subtypes, control mice accumulating either Aβ plaques (*APP;PS1* mice) or tau tangles (*Tau4RΔK* mice) were also profiled. Using scRNA-Seq approaches, we found that microglia respond to the development of Aβ and tau pathologies in a disease-stage-specific manner. During early-stage disease in 6-month-old *Tau4RΔK-AP*, but not *APP;PS1* or *Tau4RΔK* mice, the presence of both Aβ and tau pathologies induced a novel microglia subtype we termed Early-stage AD-Associated Microglia (EADAM), which is distinct from DAM and express multiple interferon-regulated genes. We found that the signature genes in EADAM were also associated with a subgroup of microglia in the brains of early (Braak II) stages of AD. In late-stage disease (12-month-old *Tau4RΔK-AP* mice), we found that another novel microglia subtype emerged in response to tau pathology that we termed Late-stage AD-Associated Microglia (LADAM), which expresses MHC and S100 family genes. We further observed a unique subtype of LADAM located near white matter, which is molecularly similar to a previously identified white matter-associated microglial subtype (WAM) that is increased during aging [25] and undergoes additional molecular transitions with Aβ and tau pathologies.

LADAM microglia, including WAM-like LADAM, were observed in the late (Braak VI), but not early (Braak II) stages of AD. Corroborating these findings, we found that sialic acid-binding immunoglobulin-like lectin (Siglec) family members are associated with specific subsets of microglia that are activated in a disease stage-specific manner in both mouse models of AD and AD patients. For example, Siglec-F is upregulated in response to Aβ pathology and is selectively expressed in Aβ-associated DAMs, while Siglec-G is upregulated in white-matter-associated LADAM in late-stage AD. These findings are consistent with a model whereby Aβ or tau pathologies stimulate the activation of microglia in different patterns. Both Aβ and tau pathologies are necessary to induce the emergence of EADAM and LADAM, and these findings provide important implications for the identification of novel molecular targets and therapeutic strategies for the treatment of AD.

## Methods

### Mice

*Tau4RΔK-AP* (*APP*^*swe*^*;PS1*Δ*E9;CamKII-tTA;TetO-TauRD*D*K*),*Tau4RΔK* (*CamKII-tTA;TetO-TauRDΔK*) and *APP;PS1* (*APP*^*swe*^*;PS1ΔE9*) transgenic mice were generated as described previously. *Tau4RΔK* were generated by crossbreeding *TetO-TauRDΔK* transgenic mice carrying mutant *Tau* fragment with regulatory element *moPrP-tetP* promoter [26] with *CamKII-tTA* mice to bring the Tau transgene under the control of the tet-off *CamKII* promoter [27]. *Tau4RΔK* mice were crossbred with *APP*^*swe*^*;PS1ΔE9* mice [28] to generate *Tau4RΔK-AP* mice that develop both Tau pathology and Aβ amyloidosis. Because of sex differences observed in *Tau4RΔK-AP* mice, only female mice were used in this study. Brains were collected from the mice at 6-month-old and 12-month-old for scRNA-Seq, histological and immunohistological studies. Among the three replicates (both 6-month-old and 12-month-old), two sets of the *APP*^*swe*^*;PS1ΔE9* and control mice also contain *CamKII-tTA* driver. Compared with the one set *APP*^*swe*^*;PS1ΔE9* and wild-type mice without *CamKII-tTA* driver, we did not observe a significant difference in microglia in our scRNA-Seq data. To suppress the potential impacts of TTA expression during postnatal development [29], mice were fed with Teklad Global 18% Protein Rodent Diet containing 200 mg/Kg doxycycline hydrochloride (Envigo Teklad Diets, Madison WI) during gestation and before weaning.

*CaMKIIα*^*CreERT2*^;*Tdp*-*43*^*F/F*^ mice with *loxP* sites flanking *Tdp*-*43* exon 3 were generated as described previously [30]. Oral tamoxifen citrate was administered in the feed (Harlan Teklad) at an average of 40 mg/kg/day for 4 weeks beginning at 6- or 9 months of age to induce recombination in excitatory forebrain neurons. Mice were singly housed during this period to monitor tamoxifen-feed intake. Afterward, the mice were returned to their original cage grouping with their littermates. Brain tissues were collected two months after the tamoxifen treatment for scRNA-Seq analysis.

All mice were housed in a climate-controlled facility (14-h dark and 10-h light cycle) with ad libitum access to food and water managed by Research Animal Resources at Johns Hopkins University. All animal procedures were in accordance strictly with the National Institutes of Health Guide for the Care and Use of Laboratory Animals and were approved by the Johns Hopkins University Animal Care and Use Committee.

### scRNA-Seq cell preparation

Mouse cortices were collected and dissociated using a previously published protocol [31, 32]. Basically, cerebral cortices and hippocampi were dissected into Hibernate-A media with a 2% B-27 and GlutaMAX supplement (0.5 mM final). Tissues were dissociated in papain (Worthington) and debris was removed using OptiPrep density gradient media following cell dissociation. Cells were then processed immediately for scRNA-Seq.

### Study design and participant

The postmortem tissues used in the present study were provided by the Johns Hopkins Brain Resource Center. For histological analysis, formalin-fixed paraffin-embedded (FFPE) tissue Sects. (10 μm) of the inferior parietal region were obtained from 36 pathologically confirmed AD cases with Braak neurofibrillary stages II-VI and controls. Details of human cases used for histology are available in Table S10. The cohort (*n* = 68) included 35 males and 33 females ages 51 to 96 years (x = 75.84), 62 Whites, and 5 African-Americans. The average postmortem interval was 23 h. We also examined FFPE hippocampal samples from patients with non-AD tauopathies for histology (Table S10).

In addition to the FFPE sections, we also examined frozen samples from the superior frontal gyrus and entorhinal cortex in AD cases for snRNA-Seq (*n* = 8), as well as 2 samples from patients with non-AD tauopathy. Details of human cases used for snRNA-Seq are available in Table S11. All AD subjects had been prospectively recruited, clinically characterized by the Johns Hopkins Alzheimer’s Disease Research Center (ADRC), and underwent neuropathologic postmortem examination excluding Lewy body disease or non-AD tauopathies. The clinical and autopsy components of this study were approved by the Johns Hopkins Medicine IRB.

### Isolation of nuclei from the frozen human brain for snRNA-Seq

Flash-frozen brain tissue was processed for snRNA-Seq following a modified 10 × Genomics protocol. Briefly, lysis buffer containing (10 mM Tris–HCl pH7.4, 10 mM NaCl, 3 mM MgCl2, 0.1% Tween-20, 0.1% Nonidet P40, 0.01% Digitonin, 1 U/ul RNase inhibitor, 1% BSA) was added into a tube containing brain micropunch (~ 100 μm) and incubated on ice for 15 min with gentle pestle grinding (5 strokes every 3 min). Wash buffer (10 mM Tris–HCl pH7.4, 10 mM NaCl, 3 mM MgCl2, 0.1% Tween-20, 0.2 U/ul RNase inhibitor, 1% BSA) was added and filtered through a 50 μm filter. Debris was removed using OptiPrep density gradient media and nuclei morphology was accessed under the light microscope. Nuclei were then immediately processed for snRNA-Seq.

### scRNA-Seq/snRNA-Seq generation

Cells or nuclei were loaded into the 10 × Genomics Chromium Single Cell System (10 × Genomics) and libraries were generated using v3.1 chemistry following the manufacturer’s instructions. Three biological replicates, where each replicates consisted of littermate mice, were used for both 6-month-old and 12-month-old scRNA-Seq runs. Two technical replicates were used for TDPKO scRNA-Seq. Two biological replicates, where each biological replicate had a technical replicate, were used for human snRNA-Seq. Libraries were sequenced on Illumina NovaSeq6000. scRNA-Seq data were first processed through the Cell Ranger (v.3.1.0, 10 × Genomics) with default parameters, aligned to the mm10 genome (refdata-cellranger-mm10-3.0.0), and matrix files were used for subsequent bioinformatic analysis. snRNA-Seq data were first processed through the Cell Ranger (v.5.0.0, 10 × Genomics) with ‘include-introns’, aligned to the GRCh38 genome (refdata-gex-GRCh38-2020-A), and matrix files were used for subsequent bioinformatic analysis.

### scRNA-Seq data analysis

#### Processing scRNA-Seq datasets

Seurat v3.15 [33] was used to process matrix files, first by selecting cells with more than 500 genes, 1000 UMI, and less than 50% mitochondrial genes and 20% ribosomal genes. Datasets were then normalized using Seurat ‘*scTransform*’ function with regressing the number of genes and UMIs using ‘*vars.to.regress*’, and Harmony v1.0 [34] was used to adjust for batch variation by treating individual scRNA-Seq run as a variance group.

The top 20 variables obtained from Harmony analysis were used for UMAP dimensional reduction. The Louvain clustering algorithm was used to first identify main cell types with default resolution and the top 20 reduction variables obtained from Harmony analysis. Individual cell types were first identified by cross-referencing previous scRNA-Seq datasets [32, 35], ASCOT database [36], as well as other datasets obtained from brain myeloid and microglia [21, 37, 38]. Doublet cells—cells that express both cell-type-specific genes (~ 5% of the overall datasets) and unhealthy cells (when mitochondrial/ribosomal genes were expressed at much higher levels compared to other clusters) were excluded from further analysis. This was performed in individual ages, across all genotypes. Individual microglia were subsetted and processed as described above. Any doublet cells (~ 2%) that couldn’t be previously identified were removed for further analysis. WAM [25], EADAM, or LADAM genes (top 50 genes from differential gene expression lists) were superimposed using Seurat *AddModuleScore* function to calculate module score.

Differential gene tests on the scRNA-Seq datasets were initially performed using Seurat v3.15’*FindAllMarkers’* function (*test.use* = *“wilcox”, logfc.threshold* = *0.5, min.pct* = *0.2)*. A cluster of microglia that was distributed almost equally among all four genotypes, and showed higher expression of immediate-early genes such as *Fos, Atf3*, and *Junb *[39], and this cluster is derived from technical artifacts during the heat-activated enzymatic dissociation step [39] and these microglia were then removed for any downstream analysis.

To identify the percentage or expression level of EADAM or LADAM genes across genotypes, as well as in other mouse models of neurodegenerative disorders and human AD samples, EADAM (Table ST3) and/or LADAM (Table ST5)-enriched genes were first identified(higher than average logfc > 0.2). Cells that express higher than UMI > 3 were flagged as positive cells, and then the percentage of positive cells relative to the total number of cells in a cluster was identified. These parameters can clearly be defined over 98% of EADAM or LADAM cells in our AD scRNA-Seq dataset.

#### Regulons

To identify regulons controlling gene expression in different microglial populations across ages and genotypes, SCENIC [40] using python implemented pySCENIC v.0.10.0 Gene regulatory networks (using –masks_dropouts), regulons and network activity of regulons was calculated using default parameters with mm10 feather files on the microglial dataset using raw count matrix. Regulon specificity scores were ranked following the SCENIC pipeline and top regulons with a z-score higher than 2 were identified as microglia-specific.

#### GO pathway analysis

Top differential genes (adjusted *p*-value < 0.05, fold change > 0.5) in each microglial cluster were used as an input for GO pathway analysis using ClusterProfiler (v3.12.0) [41].

#### RNA velocity

RNA velocity [42] was utilized to understand the dynamic state of microglia, and how each genotype-specific disease-associated microglia cluster was initiated across AD progression. Kallisto v0.46.2 and bustools v2.27.9 [43, 44] python wrapper kb-python were used to obtain spliced and unspliced transcripts using –lamanno with GRCm38 mouse genome. Scanpy v1.5.1 [45] and scVelo v0.2.1 [46] were used to process the Kallisto output with default parameters, based on UMAP coordinates obtained from Seurat.

#### Pseudotime analysis

Monocle v3.0.2 [47] was used to perform pseudotime analysis to identify differences in gene expression across microglial disease states, where the trajectory routes used for pseudotime analysis were identified based on trajectories from RNA velocity analysis, and high-variance genes (q < 0.001) were used for pseudotime plotting.

### Histology and Immunohistochemical analysis

For the histological and immunohistochemical analysis, mice were anesthetized and brains were removed and weighed. Hemibrains were fixed by submerging into 4% PFA in PBS, embedded into the paraffin, and sectioned in the sagittal plane. For histological analyses, 10 μm brain sections were stained with hematoxylin and eosin (H&E) or Cresyl violet. For immunohistochemical analysis, sections were treated with 10 mM citrate buffer (pH 6.0) by microwave in high power for 6 min for antigen retrieval; endogenous peroxidase was quenched by treating with 0.3% H_2_O_2_, and nonspecific binding of antibodies was eliminated using blocking buffer (10% normal goat serum in PBS with 0.3% Triton-X) for one hour at room temperature. The primary antibody was prepared in a blocking buffer and was applied overnight at 4 °C, followed by a secondary antibody for 30 min incubation at room temperature. For the secondary antibody and avidin-biotinylated peroxidase system, we used the Vectastain Universal Elite ABC kit (Vector Laboratories). Brain sections were stained with: antiserum against Aβ peptides 6E10 (1:1,000; SIG-39300, Covance); rabbit antiserum against phosphorylated S422 of tau (1:2,000; 44764G, Invitrogen, Carlsbad, CA); polyclonal antiserum against GFAP (1:1,000, Z0334, Dako Corporation, Carpinteria, CA); polyclonal antiserum against microglial (IBA1, 1:1,000, CP290, Biocare Medical, CA); monoclonal antibody against microglial (IBA1, 1:1000, MA5-27,728), monoclonal antibody against NeuN (MAB377, Millipore); polyclonal antiserum against Siglec-10 (1:50, HPA027093, Sigma); monoclonal Antibody Siglec-F (CD170, 1:300, 14–1702-82, ThermoFisher Scientific), polyclonal antiserum against microglial CD22 (Cy34.1, 1:200, BE0011, BioCell); monoclonal antibody against LPL (1:200, ab21356, Abcam); monoclonal antibody against Lgals3 (1:200, 14–5301-82, Invitrogen). monoclonal antibody against STAT1 (1:200, AHO0832 ThermoFisher Scientific).

For immunofluorescence, the section were detected by secondary antibodies: Alexa Flour 488 goat anti-rabbit IgG(H + L), Alexa Flour 488 goat anti-mouse IgG(H + L); Alexa Flour 488 goat anti-rat IgG(H + L); Alexa Flour 594 goat anti-rabbit IgG(H + L), or Alexa Flour 594 goat anti-rabbit IgG(H + L) (ThermoFisher Scientific). Sections were counterstained with DAPI before being examined under a Zeiss LSM 510 laser scanning fluorescence confocal microscope. Z-stack projections were made from serial scanning every 0.34 μm to reconstruct the images.

Thioflavin-T (ThioT) staining, after deparaffinized and hydrated, slides were incubated in potassium permanganate solution (0.25%) for 5 min, then the slides were transferred to 1% potassium-disulfate and oxalic acid for 5 min before incubated in 0.02% ThioT for 8 min. Slides were dehydrated and mounted with an aqueous mounting medium for fluorescence.

The quantitative score of Siglec-10 positive cells in the inferior parietal region of human brains was the average count of at least three brain sections in a power (2,000X) microscope field using the ImageJ program. Siglec-10 signals in gray and white matter in the same section were counted separately. The histological section of the human inferior parietal region includes the cerebral cortex and subjacent white matter. These two compartments can be easily separated by their cytological features. The cortex contains a large number of neurons characterized by their large size and prominent nucleus and nucleolus. By contrast, the white matter is rich in oligodendrocytes, which are small, have a compact nucleus and virtually no cytoplasm; and astrocytes. Neurons are virtually absent in the white matter.

Neurons in mouse brains were identified by NeuN staining. The quantitative scores of NeuN + , IBA1 + , SiglecG + , Lgal3 + cells were the average counts in a power (2,000X) microscope field of at least three sagittal sections at 2 mm from the midline of the mouse brains using ImageJ.

To quantify the Amyloid plaques, at least three sagittal sections at 2 mm from the midline of the mouse brains were selected. Amyloid plaque size, numbers, or area fraction were quantified based on the 6E10 immunoreactivity or ThioT staining using ImageJ.

### Statistical analysis

All data were analyzed statistically by unpaired Student’s two-tailed t-test or one-way ANOVA with Tukey correction for multiple comparisons for cell counting analysis, and two-way ANOVA with Tukey’s multiple comparison test for scRNA-Seq cell distribution analysis (distribution of microglia clusters within each genotype and distribution of cell types within each genotype), using GraphPad Prism (GraphPad Software, La Jolla CA the USA). In all tests, values of *p* < *0.05* were considered to indicate significance.

## Results

### ScRNA-Seq of microglia in mice harboring Aβ plaques and/or tau deposition

To identify microglia subtypes through AD-like pathology progression, we performed scRNA-Seq in the cerebral cortex and hippocampus of transgenic mice displaying Aβ plaques and/or tau pathologies. We took advantage of our previously characterized mouse model of AD (*Tau4RΔK-AP* mice), which exhibits AD-like pathologies including Aβ plaques and tau tangles and results in progressive neuronal loss and brain atrophy [24]. In a cross-breeding strategy using mutant *APP*^*swe*^*;PS1ΔE9* (*APP;PS1*) [28] and *Tau4RΔK* mice, a cohort of *Tau4RΔK-AP* mice was generated. Female mice were aged to either 6-month-old or 12-month-old. Six-month-old *Tau4RΔK-AP* mice mimic an early AD stage, characterized by low levels of Aβ plaques with minimum tau phosphorylation (Figure S1) and deposition [24] in the hippocampus but not in the cortex and no loss of neurons (Figure S1). Twelve-month-old *Tau4RΔK-AP* mice mimic a late disease stage, characterized by robust Aβ plaques (Figure S1), tau deposition (Figure S1), loss of neurons and brain atrophy (Figure S1). For these two time points, in addition to *Tau4RΔK-AP* mice, we also collected cerebral cortices and hippocampi from littermate controls (*WT)*, *APP;PS1* (Aβ plaques (S1)), and *Tau4RΔK* (tau deposition, neuronal loss (Figure S1), and brain atrophy (Figure S1)) mice. Compared to *Tau4RΔK* mice at 12 months of age, Aβ plaques accelerated tau pathogenesis and tau aggregation-dependent neuronal loss and brain atrophy (Figure S1) in 12-month-old *Tau4RΔK-AP* mice, as previously shown [24]. On the other hand, tau aggregation has little effect on the deposition of Aβ plaques, as well as plaques morphology, in *Tau4RΔK-AP* mice as compared with that of *APP;PS1* mice (Figure S1). Twelve-month-old *Tau4RΔK-AP* mice also showed an increase in plaque numbers and areas compared to 6-month-old *Tau4RΔK-AP* mice but plaque size did not differ (Figure S1). We also observed an increase in microglia in the cerebral cortex, hippocampus, and corpus callosum between 6 and 12 months (Figure S2). We then subjected these tissues across 4 genotypes at both 6 months and 12 months to scRNA-Seq analysis.

We first analyzed female 6-month-old cerebral cortices and hippocampus, across four genotypes in triplicates (Figure S3). We observed all major cell types of CNS, including neurons, astrocytes, oligodendrocytes, and microglia (Figure S3, Table S1). Previously, we confirmed the initiation of tau deposition in 6-month-old cerebral cortices [24]. We observed the absence of neuronal loss or brain atrophy in these mice, along with the presence of Aβ plaques (Figure S1). As expected, we observed a few DAM microglia in *Tau4RΔK-AP* brains in early-stage disease (Figures S3, S9). Analyzing three biological replicate samples, we observed that microglia in *APP;PS1* and *Tau4RΔK-AP* mice (Figures S3, S5), expressed a high level of disease-associated microglia 1 (DAM1) marker including *Cst7* (Figure S3). However, we failed to observe DAM2 markers, including *Gpnmb*, even in *APP;PS1* and *Tau4RΔK-AP* mice at this age (Figure S3).

Using scRNA-Seq, we also identified cell clusters corresponding to major subtypes of neurons, glia, and immune cells (Figure S4, Table S2) in 12-month-old mice of all four genotypes, analyzing three biological replicates. Consistent with our previous observation, AD-like pathologies in *Tau4RΔK-AP* mice led to gliosis and neuronal loss. An increase in the microglia population along with a corresponding decrease in the number of neurons was observed in the *Tau4RΔK-AP* mice (Figures S1, S4). Compared to the other three control mice (*WT*, *APP;PS1,* and *Tau4RΔK* mice), *Tau4RΔK-AP* mice also showed an increase in immune cells as well (Figure S4).

*APP;PS1*, *Tau4RΔK* and *Tau4RΔK-AP* mice showed a marked increase in the number and proportion of microglia as compared to *WT* mice, (Figures S2, S4, S5). Known DAM1 marker genes such as *Cst7*, and DAM2 genes such as *Gpnmb*, were strongly expressed in both *APP;PS1* and *Tau4RΔK-AP* mice (Figure S4). These results establish that these scRNA-Seq datasets have sufficient quantitative power to identify microglia subtypes that are influenced by Aβ and/or tau deposition in a disease stage-specific manner.

### Identification of a novel Early-stage AD-Associated Microglia (EADAM) induced by both Aβ plaques and tau deposition

**Fig. 1:**
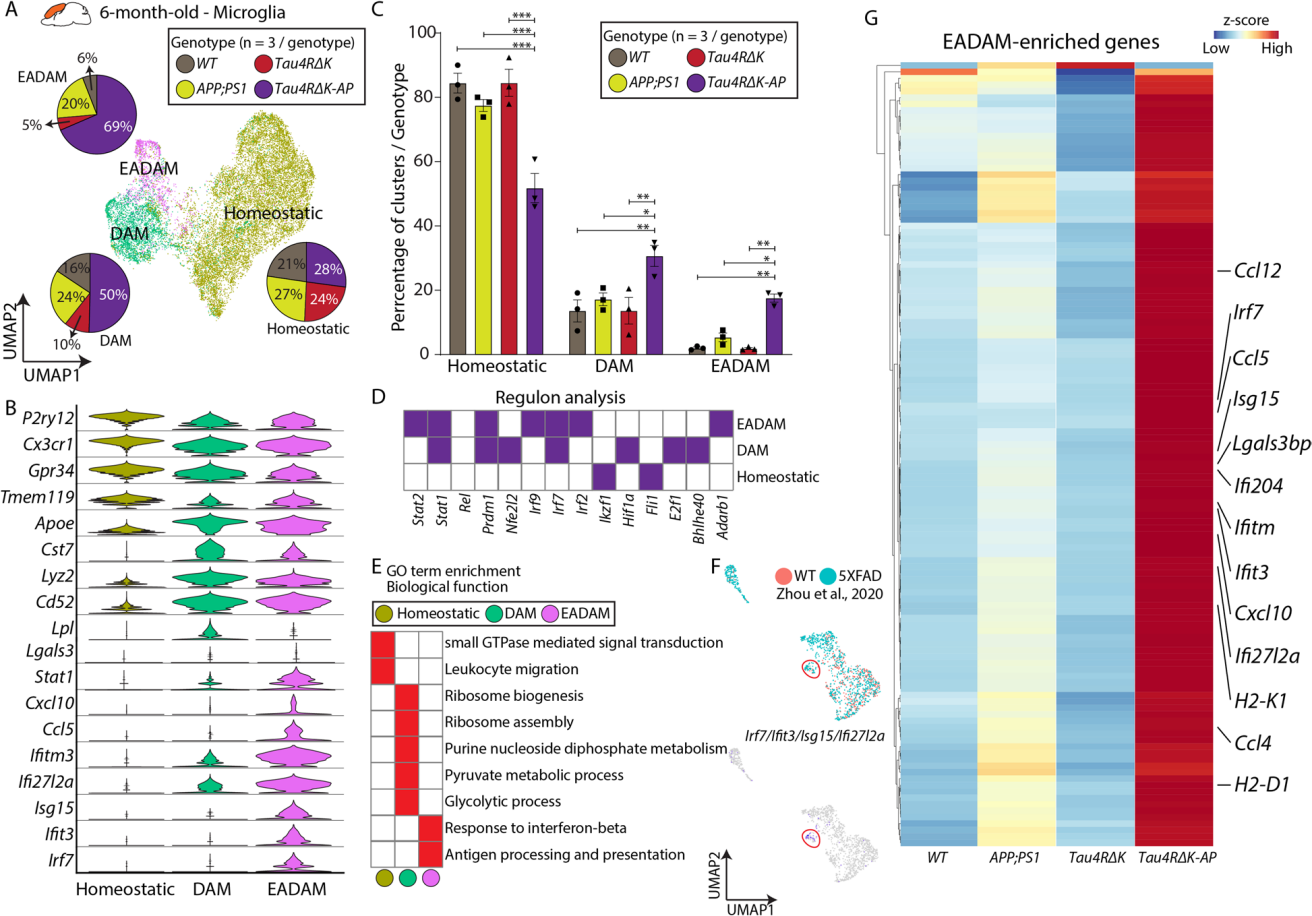
The microglia subtype EADAM is observed in the *Tau4RΔK-AP* mice in the 6-month-old cortex. **A** UMAP plot showing microglia clusters—Homeostatic, DAM, and EADAM, across 4 genotypes in 6-month-old cortex (*n* = 3/genotype), and pie graphs showing the distribution of 3 microglia clusters across genotypes (*n* = 3/genotype). **B** Violin plots showing top cluster genes of 3 microglia clusters. **C** Bar graphs showing the distribution of 3 microglia clusters within each genotype (*n* = 3/genotype). Note the significant increase in the EADAM cluster in *Tau4RΔK-AP* mice. **D** Regulon analysis with SCENIC [40], showing key regulons in each microglia cluster. **E** GO analysis of top differential genes reveals a biological function in each microglial cluster. **F** UMAP plots of 7-month-old 5XFAD mice in Zhou et al. 2020 [23] (top) and a small cluster of cells (red circle) expressing a few EADAM-enriched markers (bottom). **G** Heatmap showing expression of EADAM-enriched genes across genotypes. * *P* < *0.05*, ** *P* < *0.005*, *** *P* < *0.0001*

We then subsetted and profiled microglia from the cerebral cortex and hippocampus of 6-month-old *Tau4RΔK-AP* mice, along with the three control mouse lines. Homeostatic microglia expressing genes such as *Tmem119* and *P2ry12*, were predominantly detected across all genotypes (Fig. 1A, B), but two additional microglia clusters were also observed (Fig. 1A, Table S3). The first microglia cluster expressed classic DAM1-like markers, including *Apoe*, *Cst7*, and *Lpl* (Fig. 1B). The second microglia cluster expressed DAM1-like markers but also displayed a unique gene module consisting of interferon-related genes, such as *Ifitm3, Ifit27l2a*, and *Ifit3*, as well as *Stat1* (Fig. 1B). Since this cluster was predominantly composed of *Tau4RΔK-AP* microglia and detected during the early and presymptomatic stage of the disease (Fig. 1C), we thus identify this cluster as Early-stage AD-Associated Microglia (EADAM). Our regulon and pathway analysis confirmed the association of EADAM with interferon-regulating transcription factor *Irf2/7/9* (Fig. 1D) and linkage to the GO term ‘Response to Interferon-beta’ (Fig. 1E).

A small EADAM-like cluster is also detected in *APP;PS1* microglia (Fig. 1A, C). This observation is consistent with the previous report that the accumulation of Aβ plaques leads to the activation of the interferon pathway [48–51]. A small cluster of cells that resemble EADAM-like microglia, expressing *Irf7* and other EADAM-enriched genes, such as *Ifit3, Isg15, Ifi27l2a*, were also detected in 7-month-old 5xFAD snRNA-Seq data [23] (Fig. 1F).

We next compared differential gene expressions between EADAM populations across four genotypes. Despite a significant contribution of *APP;PS1* microglia to EADAM cluster, the overall level of EADAM-enriched genes was expressed at a much lower level in *APP;PS1* microglia than that of *Tau4RΔK-AP* EADAM (Fig. 1F, Table S4). On the other hand, the expression of interferon pathway-related genes was not observed in *Tau4RΔK* microglia, suggesting that while the accumulation of Aβ plaques leads to activation of the interferon pathway, both Aβ plaques and tau aggregation are necessary to fully induce EADAM gene modules in *Tau4RΔK-AP* microglia. In summary, our data identify a novel microglia subtype termed EADAM that is induced by a combination of Aβ plaques and tau deposition during early-stage AD.

### Identification of Late-stage AD-Associated Microglia (LADAM)

**Fig. 2:**
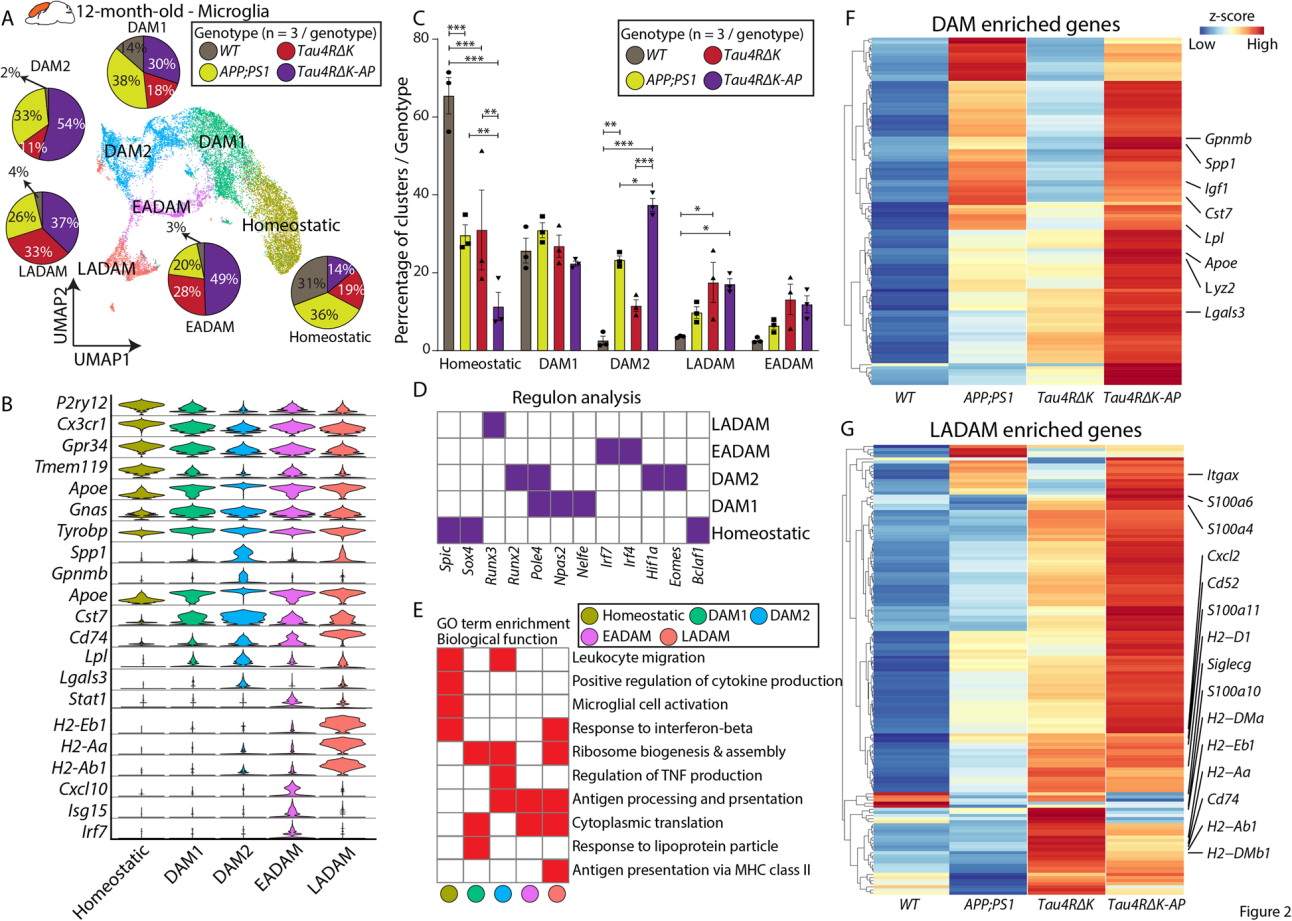
The microglia subtype LADAM is observed in the *Tau4RΔK-AP* mice in the 12-month-old cortex. **A** UMAP plot showing microglia clusters—Homeostatic, DAM1, DAM2, EADAM, and LADAM, across 4 genotypes in 12-month-old cortex (*n* = 3/genotype), and pie graphs showing the distribution of 4 microglia clusters across genotypes (*n* = 3/genotype). **B** Violin plots showing top cluster genes of 3 microglia clusters. **C** Bar graphs showing the distribution of 5 microglia clusters within each genotype (*n* = 3/genotype). Note the significant increase in the LADAM cluster in *Tau4RΔK-AP* mice. **D** Regulon analysis with SCENIC [40], showing key regulons in each microglia cluster. **E** GO analysis of top differential genes reveals a biological function in each microglia cluster. **F** Heatmap showing expression of DAM-enriched genes across genotypes. **G** Heatmap showing expression of LADAM-enriched genes across genotypes. * *P* < *0.05*, ** *P* < *0.005*, *** *P* < *0.0001*

To further clarify the influence of Aβ plaques and tau tangles in microglia pathophysiology during late-stage AD, we unbiasedly subdivided subsetted microglia populations into five distinguishable clusters across all four genotypes (*WT*, *APP;PS1, Tau4RΔK*, and *Tau4RΔK-AP*) in 12-month-old mice. A few notable differences were observed in 12-month-old mice compared to 6-month-old mice. The DAM cluster was further divided into two clusters—DAM1 and DAM2. The DAM1 cluster expressed genes such as *Tyrobp, Ctsd, C1qa* (Fig. 2A, B, Table S5)*,* whereas DAM2 expressed DAM1-enriched genes and additional disease-related genes, such as *Gpnmb*, *Cst7*, and *Spp1* (Fig. 2B, Table S5). The induction of DAM2 in response to Aβ plaques has been documented in a variety of APP-based mouse models of AD [20]. Indeed, both clusters were highly represented in animal models with Aβ pathologies (*APP;PS1* and *Tau4RΔK-AP* mice, Fig. 2A, C), but the DAM2 abundance in mouse models with only tau pathologies (*Tau4RΔK* mice) was low (Fig. 2A, C, Table S6). These observations are consistent with the view that these DAM clusters arise mainly as a result of Aβ plaques. Similar to situations in the EADAM population of 6-month-old mice, *Tau4RΔK-AP* mice generally have higher levels of DAM-enriched genes than that of *APP;PS1* mice (Fig. 2F). Since Aβ plaques level in *Tau4RΔK-AP* mice is similar to that of *APP;PS1* mice (Figure S1), this observation indicates that while Aβ plaques can induce DAM2, tau deposition and/or neuronal loss could further enhance DAM-enriched genes in *Tau4RΔK-AP* mice.

A diminished EADAM cluster was still detected in 12-month-old *Tau4RΔK-AP* mice. Interestingly, EADAM-like microglia were also detected in 12-month-old *Tau4RΔK* mice, although they were absent at 6 months of age (Fig. 2C). In addition to the widespread tau aggregation, *Tau4RΔK* mice already show a neuronal loss at 12-month-old [24], suggesting that while the presence of tau aggregation alone may not induce EADAM formation, cell death-induced inflammation may also induce EADAM-like gene expression in microglia.

We also observed a unique microglia cluster that was not detected in 6-month-old mice (Fig. 2A). Cells in this cluster expressed MHC Class II genes, such as *Cd74*, *H2-A2*, *H2-Eb1*, and *H2-Ab1*, and additional markers like *Lgals3* (Fig. 2B, Table S4). We termed these microglia Late-stage AD-Associated Microglia (LADAM), which were found in *Tau4RΔK-AP*, as well as *Tau4RΔK*, and *APP;PS1* samples (Fig. 2A, C). Regulon analysis identified key transcription factors associated with gene regulatory networks across all microglial clusters. Notably, while *Hif1a* and *Eomes* are selectively expressed in the DAM2, *Runx3* and *Irf4/7* are expressed in LADAM (Fig. 2D), and GO analysis on LADAM shows enrichment for ‘MHC Class II’ (Fig. 2E). In addition to MHC class II genes, we also identified enriched expressions of *S100a* family genes, including *S100a4, S100a6*, and *S100a10* (Fig. 2G).

Although LADAM was observed in *Tau4RΔK-AP*,*Tau4RΔK*, and *APP;PS1* samples, we found that many LADAM-specific genes (e.g. MHC Class II genes) are more highly expressed in *Tau4RΔK* than in *APP;PS1* microglia (Fig. 2G), suggesting that this microglia subtype might be induced by the development of tauopathies, such as tau tangles or tau phosphorylation. Moreover, relative to *Tau4RΔK* microglia, we found that LADAM gene signatures were more pronounced in *Tau4RΔK-AP* microglia (Fig. 2G, Table S5), which exhibited Aβ plaques, more robust tau pathologies and neuronal loss. These datasets show that tau pathologies are sufficient to induce the emergence of LADAM and that their amplification is Aβ plaque-dependent. However, since MHC class II genes that are expressed in LADAM, such as *Cd74*, *H2-A2*, *H2-Eb1*, and *H2-Ab1*, have also been previously observed in microglia in both the *CK-p25* neurodegeneration mouse model [52, 53] and some mouse models of late-stage amyloidosis [48–50], some of LADAM gene modules may be induced at least in part by neuronal death.

### Requirement of both Aβ plaques and tau deposition to elicit the emergence of disease stage-dependent microglia subtypes

**Fig. 3:**
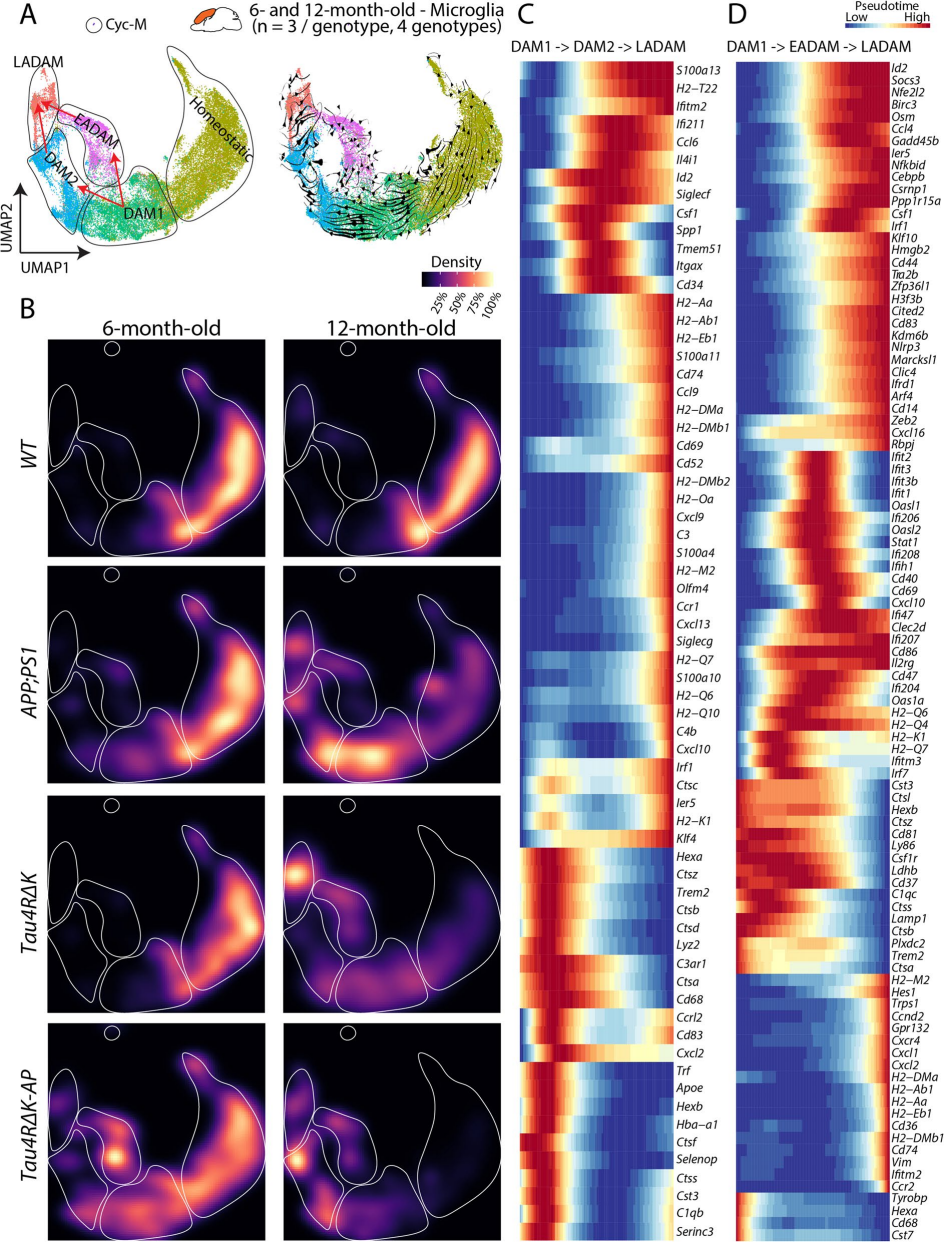
The emergence of disease stage-dependent microglia subtypes coincides with Aβ and tau deposition. **A** UMAP plot showing microglia clusters in the 6- and 12-month-old cortex (all genotypes) (left) and UMAP plot with RNA velocity (right) showing 2 potential transitions between DAM1 and LADAM, with transitions into EADAM and/or DAM2 in between. **B** UMAP plot showing the density of the captured microglia clusters across genotypes in the 12-month-old cortex. **C** Pseudotime analysis showing gene expression changes between DAM1, DAM2, and LADAM. **D** Pseudotime analysis showing gene expression changes between DAM1, EADAM, and LADAM

To clarify how the emergence of EADAM, DAM2, and LADAM is regulated by Aβ plaques and tau deposition, we merged datasets obtained from microglia of both 6-month-old and 12-month-old samples (Fig. 3A). RNA velocity analysis shows that DAM1 is likely to give rise to, or to be closely related to, both DAM2 (Aβ plaques driven) and EADAM (Aβ plaque and tau deposition driven), whereas the convergence of DAM2 (Aβ plaque-driven and tau deposition-enhanced) and EADAM may ultimately lead to induction of LADAM, as the result of tau deposition/cell loss-driven and Aβ plaque-formation (Fig. 3B). As shown above, EADAM is composed mostly of cells derived from 6-month-old *Tau4RΔK-AP* mice (Fig. 3B). The DAM2 clusters were enriched in 12-month-old *APP;PS1*, and *Tau4RΔK-AP* mice (Fig. 3B), and LADAM clusters were enriched in 12-month-old *APP;PS1*, *Tau4RΔK*, and *Tau4RΔK-AP* mice (Fig. 3B).

Furthermore, RNA velocity coupled with pseudotime analysis (Fig. 3C, D) identified gene expression changes occurring during the temporal progression from DAM1 to DAM2 to LADAM, with changes in multiple inflammation-related genes, such as *Ccl6, Csf1, Cd34* (Fig. 3C); as well as during the temporal progression from DAM1 to EADAM to LADAM, with changes in interferon genes, such as *Ifiti* family members (Fig. 3D). We have also identified NF-kB pathway genes, which are important in tau seeding and spreading, are enriched in EADAM and *Tau4RΔK-AP* mice at 6-month-old (Figure S6) as previously shown [54].

A microglia cluster(s) that express either interferon genes and/or MHC Class II genes have been identified in other neurodegenerative models, such as *CK-p25* [53] and Trem2 deficient [55, 56] FTDP-17-linked tau model (P301L) [57] crossed with *PS2APP* [58] mice. However, none of the disease models showed the development of separated EADAM and LADAM clusters in a stage-dependent manner. This led to the hypothesis that, while potential conservation of EADAM- or LADAM-like subtypes might occur across both neurodegeneration and neuroinflammation, a combination of both Aβ plaques and tau deposition is critical for the induction of disease-stage specific microglia subtypes in AD.

**Fig. 4:**
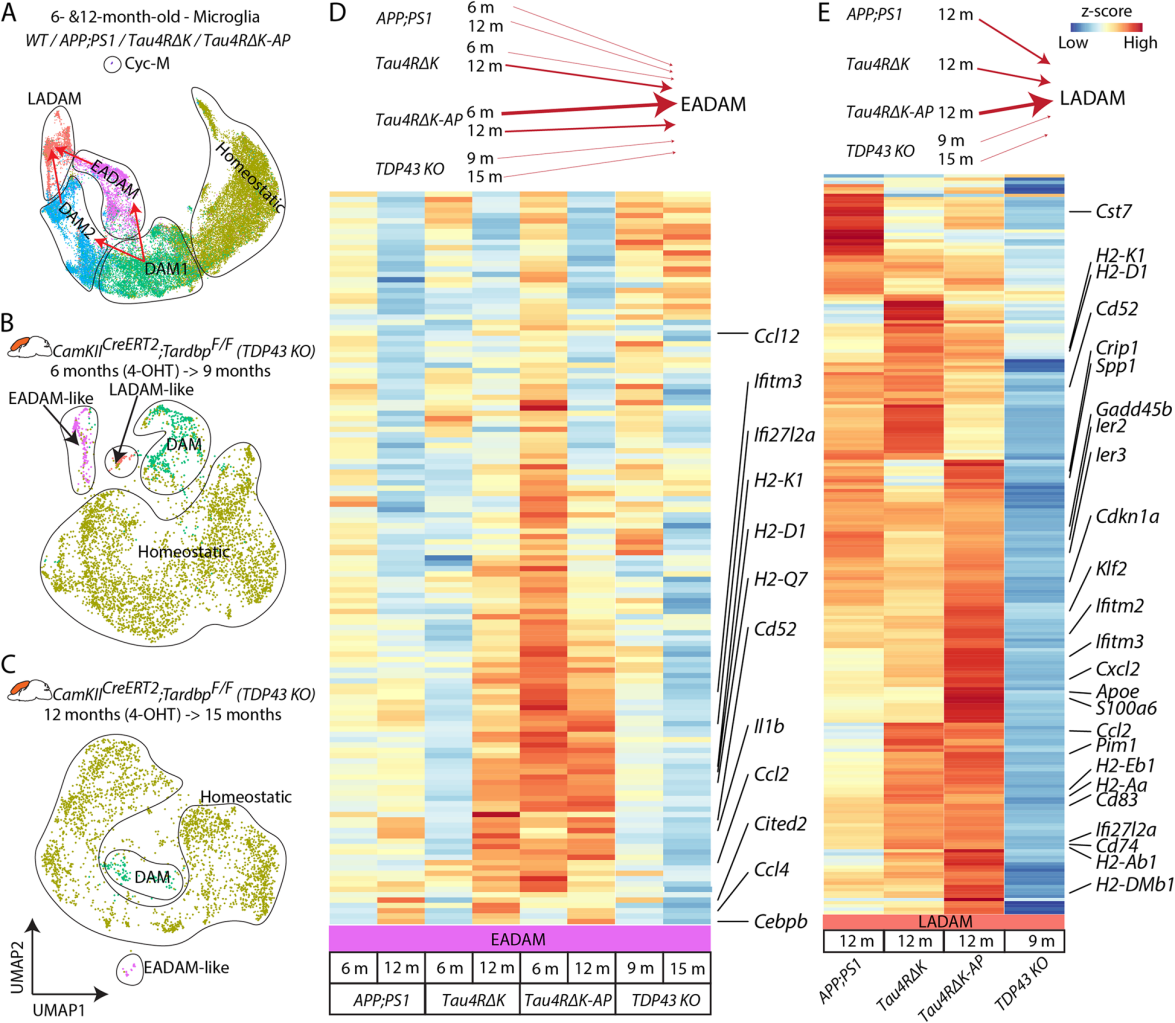
Analysis of EADAM and LADAM markers in other mouse models of neurodegenerative disease. **A** UMAP plot showing microglia clusters in the 6 and 12-month-old cortex (all genotypes) (Fig. 3A left panel). **B** UMAP plot showing microglia clusters in *CamKII*^*CreERT2*^*;Tardbp*^*lox/lox*^ (*TDP43 KO*), 4-OHT induction at 6 months and collected at 9 months. *N* = 2. **C** UMAP plot showing microglia clusters in *CamKII*^*CreERT2*^*;Tardbp*^*lox/lox*^ (*TDP43 KO*), 4-OHT induction at 12 months and collected at 15 months. *N* = 2. **D** Heatmap plot showing expression of EADAM-enriched genes across AD genotypes and in *TDP43 KO*. **E** Heatmap plot showing expression of LADAM-enriched genes across AD genotypes and in *TDP43 KO*

To test this hypothesis, we then used another neurodegenerative mouse model *CamKII*^*CreERT2*^*;Tardbp*^*lox/lox*^* (TDP43 KO*), where a tamoxifen induction leads to conditional deletion of TDP-43 in the forebrain of the mice, resulting in the selective vulnerability of hippocampal CA3 neurons [30]. We generated scRNA-Seq data from the cerebral cortex and hippocampus of 2 different *TDP43 KO* samples, one being induced with 4-OHT at 6 months and collected at 9 months (*TDP43 KO*^6−>9^) and the other being induced with 4-OHT at 12 months and collected at 15 months (*TDP43 KO*^12−>15^) (Fig. 4A-C). While both *TDP43 KO* mice showed DAM1-like clusters, *TDP43 KO*^6−>9^ showed both EADAM- and LADAM-like clusters that respectively selectively express interferon genes or MHC Class II genes (Fig. 4A-C, Table S7). *TDP43 KO*^12−>15^ also showed an EADAM-like cluster (Fig. 4A-C, Table S8), supporting the view that EADAM- or LADAM-like subtypes might occur across neurodegenerative.

We then integrated datasets from AD mice with those from *TDP43 KO* mice (Fig. 4D, E). As mentioned above, both 6-month-old *APP;PS1* and *Tau4RΔK-AP* microglia showed a similar expression pattern of EADAM-enriched genes (Fig. 4D), but *Tau4RΔK-AP* microglia displayed much higher expression levels of these genes than did *APP;PS1* microglia (Fig. 4D). 12-month-old *Tau4RΔK* and *Tau4RΔK-AP* microglia showed overall lower expression levels, but broadly similar EADAM expression patterns (Fig. 4D). Both *TDP43 KO* microglia showed a much weaker level of EADAM-enriched genes, similar to the 6-month-old *APP;PS1* microglia (Fig. 4D).

LADAM-enriched genes are composed of DAM2-enriched genes, MHC Class II genes, and *S100a* family genes. While the LADAM-like cluster in *TDP43 KO*^6−>9^ microglia expressed MHC Class II genes, the level of expression was much lower than any of *APP;PS1, Tau4RΔK* and *Tau4RΔK-AP* microglia (Fig. 4E). We also failed to detect any DAM2-like clusters, or either *S100a* family genes and *Siglecg* in *TDP43 KO*^6−>9^ microglia.

A similar observation was also made in *CK-p25* microglia [53] (Figure S6). While the integration of this dataset was not possible due to the use of different single-cell formats (SMART-Seq was used to generate the *CK-p25* dataset), we noted that both EADAM-like interferon genes and LADAM-like MHC Class II genes were more highly expressed in *CK-p25* microglia than the control (Figure S7). However, both EADAM-like and LADAM-like microglia were intermingled, rather than forming separate clusters. We also failed to detect any *S100a* family genes and *Siglecg* expression in the *CK-p25* microglia dataset, similar to our observations in the *TDP43 KO* dataset (Figure S7).

This indicates that Aβ plaque or tau pathology independently induces specific gene signatures in microglia like EADAM and LADAM, and similar microglia clusters can also be observed in other neurodegeneration models. Neuroinflammation and/or cell loss that occurs in neurodegeneration diseases could be a potential trigger for microglia to develop EADAM- or LADAM-like gene expression profiles. However, the combination of both Aβ plaque and tau pathology results in separated clusters of EADAM and LADAM at different stages of the disease, much higher expression levels of the signature genes, and induction of additional molecular markers in LADAM.

We have also compared our dataset with previous findings from human snRNA-Seq [59], which have identified gene expression profiles that are associated with AD pathology. The two main identified populations from the previous study were AD1-microglia, which show a strong correlation with Aβ, and AD2 microglia, which show a correlation with tau-loading. We compared the enriched genes of AD1- and AD2-microglia onto our dataset (Figure S8), and we observed a strong correlation of AD1-enriched genes in *APP;PS1* and *Tau4RΔK-AP* at 6 months, and a strong correlation of AD2-enriched genes in *Tau4RΔK* (Figure S8).

Furthermore, we performed immunostaining with various markers of microglia subtypes which we have identified from our scRNA-Seq dataset. To first confirm the DAM activation, we stained the brain slices with LPL, an AD risk factor identified as a DAM-enriched gene (Fig. 1). LPL-positive microglia are detected in *APP;PS1* and *Tau4RΔK-AP* mice at 6 months of age and the signals are further increased at 12 months of age (Figure S9). On the other hand, no LPL signals were detected in *Tau4RΔK* and *WT* mice, suggesting that the DAM were more specifically activated by Aβ pathologies in agreement with previous findings. A similar pattern of activation was also detected in CD22 (Siglec-2) (Figure S9).

One of the most upregulated genes that are strongly related to microglial DAM phenotype is Lgals3 (Figs. 1, 2), a carbohydrate-binding protein [53, 60–62]. Homeostatic microglia do not express Lgals3 (Figs. 1, 2) [20, 60], but Lgals3 expression is significantly upregulated in microglia in response to Aβ plaques both in humans and mice [63, 64]. Furthermore, Lgals3 is associated with activated microglia, and Lgals3 is mainly expressed and released in the damaged brain by reactive microglia [65]. To further confirm the DAM activation in AD mouse models, we immunostained brain slices with anti-Lgals3 antibodies (Figure S10, S11). While Lgals3 was enriched around the Aβ plaques in *APP;PS1* and *Tau4RΔK-AP* mice (Figures S10, S11), we have also observed robust Lgals3 staining at the corpus callosum in *Tau4RΔK*, with much stronger expression in *Tau4RΔK-AP* mice (Figures S10, S11). The Lgals3 signal was co-localized with microglia marker IBA1, but not astrocyte marker GFAP, supporting the notion that Lgals3 was specifically activated in microglia by AD pathologies. We have identified a robust *Lgals3* expression in LADAM in 12-month-old *Tau4RΔK* mice (Fig. 2), and Lgals3 levels were associated with the severity of tau aggregation (Figure S11), suggesting that Lgals3 could also be induced by Tauopathy in a sub-population of LADAM that reside near the corpus callosum.In addition, we have validated that EADAM marker, STAT1, is robustly expressed in microglia in *Tau4RΔK-AP* mice at 6-month-old and 12-month-old (Figure S12), but not expressed in astrocytes, which is consistent with our scRNA-Seq finding (Fig. 1). STAT1 was not strongly expressed in glial cells in wild-type mice, *APP;PS1* and *Tau4RΔK* mice, indicating that neither Aβ nor tau pathology alone could strongly induce the STAT1 expression.

### Identification of stage-specific EADAM and LADAM signatures in AD samples

**Fig. 5:**
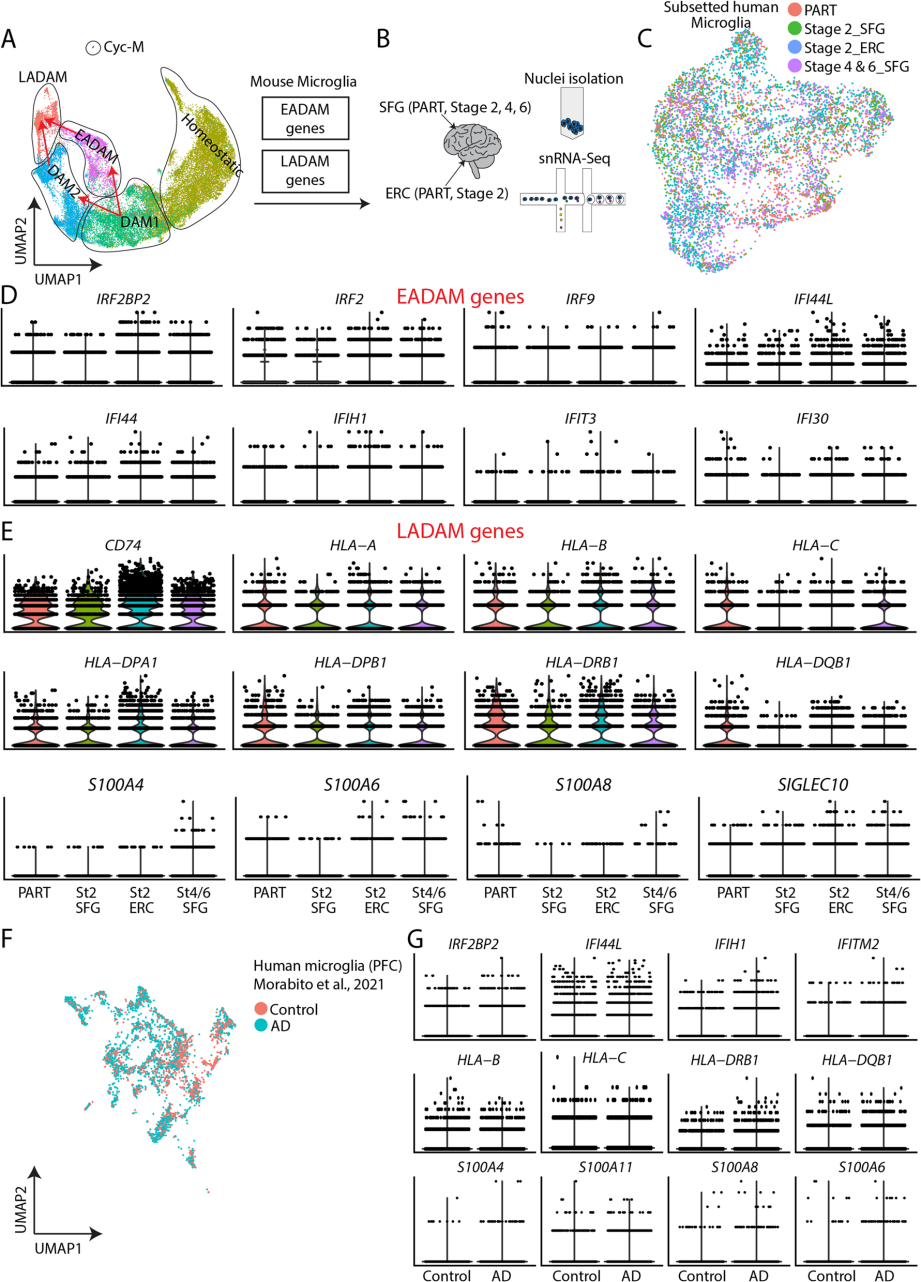
6-month-old EADAM at and 12-month-old LADAM clusters in *Tau4RΔK-AP* mice resemble microglial subtypes seen in snRNA-Seq from AD samples. **A** UMAP plot showing microglia clusters in the 6 and 12-month-old cortex (all genotypes) from Fig. 3A. EADAM and LADAM-enriched gene homologs were studied in human snRNA-Seq. **B** Schematic showing snRNA-Seq from human postmortem samples, primary age-related tauopathy (PART) entorhinal cortex (ERC), superior frontal gyrus (SFG); Stage 2 ERC, SFG; Stage 4 SFG; Stage 6 SFG. **C** UMAP plot showing the distribution of subsetted microglia from human snRNA-Seq. *N* = 2 (PART, 1102 cells), *n* = 2 (Stage 2 SFG, 750 cells), *n* = 2 (Stage 2 ERC, 2645 cells), *n* = 4 (Stage 4 and 6 SFG, 1692 cells). **D** Violin plots showing expression of EADAM-like interferon family members. **E** Violin plots showing expressions of LADAM-like MHC and S100 family members. **F** UMAP plot showing microglia from human snRNA-Seq dataset from Morabito et al., 2021. *N* = 7 (Control, 1384 cells), *n* = 11 (AD, 2742 cells). **G** Violin plots showing IFN, MHC, and S100 family members in the dataset from Morabito et al., 2021

We next generated snRNA-Seq dataset from the human superior frontal gyrus (SFG) at Braak stage 2, which is devoid of tau pathology, and entorhinal cortex (ERC) at Braak stage 2, where AD pathology is first detected and shares a pathological resemblance to our 6-month-old *Tau4RΔK-AP* mice (Fig. 5A-C). We also generated snRNA-Seq from the superior frontal gyrus (SFG) at Braak stages 4 and 6, which represent late stages of AD and share a pathological resemblance to the 12-month-old *Tau4RΔK-AP* mice. As a control, we generated a snRNA-Seq dataset from the ERC and SFG of a non-AD neurodegenerative disorder, primary-age-related-tauopathy (PART) (Fig. 5A-C).

We initially checked the expression pattern of EADAM- and LADAM-enriched gene homologs in the human snRNA-Seq dataset (Figure S13), and observed moderate conservation in the gene expression pattern. Most EADAM-enriched genes were seen at Braak stage 2 SFG and ERC (Figure S13), while LADAM-enriched genes were seen at Braak stage 4 and 6 SFG (Figure S13). Some inconsistency of expression patterns might be due to the absence of AD-specific microglia clusters in the human snRNA-Seq dataset, despite having a high resolution of 6,000 cells. This observation has been previously shown in other human snRNA-Seq datasets from brains affected by AD or other neurodegenerative diseases [66–68]. The difference between the human and animal models might be due to the fact that AD and other neurodegenerative diseases typically progress slowly, over many years, and all microglia populations may eventually end up transitioning to disease-associated states. This timing is different in virtually all animal models that were designed to undergo neurodegeneration much more rapidly.

While the presence of EADAM- and LADAM-like microglial subtypes in AD demonstrated important similarities with our mouse models, we also wanted to analyze the expression of MHC and interferon-induced genes. We observed consistent changes in the expression of interferon-regulated genes. These include the transcription factors and co-regulators *IRF2BP2*, *IRF2*, and *IRF9*, which showed higher expression at Braak stage 2 ERC than SFG, and whose expression levels were decreased in Braak stage 4/6 SFG (Fig. 5D). There also was a similar trend in the expression of other interferon-regulated genes, including *IFI44*, *IFI44L* (Fig. 5D).

We also observed a similar trend in MHC genes, such as *HLA-A*, *HLA-B*, and *HLA-DPA1*, which showed a higher expression level in PART ERC and SFG, Braak stage 2 ERC, and in Braak stage 4/6 SFG than Braak stage 2 SFG (Fig. 5E). More importantly, *S100* genes, such as *S100A4*, *S100A11*, were exclusively detected in Braak stage 4/6 SFG but were not detected in PART or other AD snRNA-Seq (Fig. 5E). This observation was also conserved in other high-quality human AD snRNA-Seq from the prefrontal cortex [66] (Fig. 5F, G), where interferon-regulated, MHC, and *S100* family genes were more highly expressed in AD samples than in the control (Fig. 5F, G). These findings thus establish the emergence of EADAM-related genes in early AD-stage and LADAM-related genes in late AD-stage and suggest that these microglia subtypes could be induced by both Aβ plaques and tau deposition in a disease stage-specific manner.

### Siglecs may serve as specific biomarkers to track Alzheimer’s disease stage-specific microglia subtypes

Our analysis identifies multiple microglial genes that are selectively expressed in late-stage AD, such as the Braak-stage-dependent expression of *SIGLEC10*, a human homolog of *Siglecg* (Fig. 5E). Since several of the Siglec family genes are thought to be relevant for AD progression [69], we focused on the expression of Siglec gene family at different microglia clusters. Homeostatic microglia expressed *Mag*, *Siglece*, *Cd33*, *Siglech* (Figure S14), and EADAM expressed similar Siglec genes as homeostatic microglia but also expressed *Siglec1* (Figure S14). Both DAM2 and LADAM clusters expressed high levels of *Siglecf* (Figure S14). Both *Siglec1* and *Siglecg* were enriched in the LADAM. Siglec-G expression was robustly detected near the corpus callosum at 12-month-old *Tau4RΔK-AP* mice (Figure S15). *Siglecf*, in particular, was enriched in both *APP;PS1,* and *Tau4RΔK-AP* mice (Figure S14) and showed increased expression in late-stage disease (Figure S14). These data suggested that Siglec genes are expressed in a pathology and/or stage-dependent manner in microglia.

**Fig. 6:**
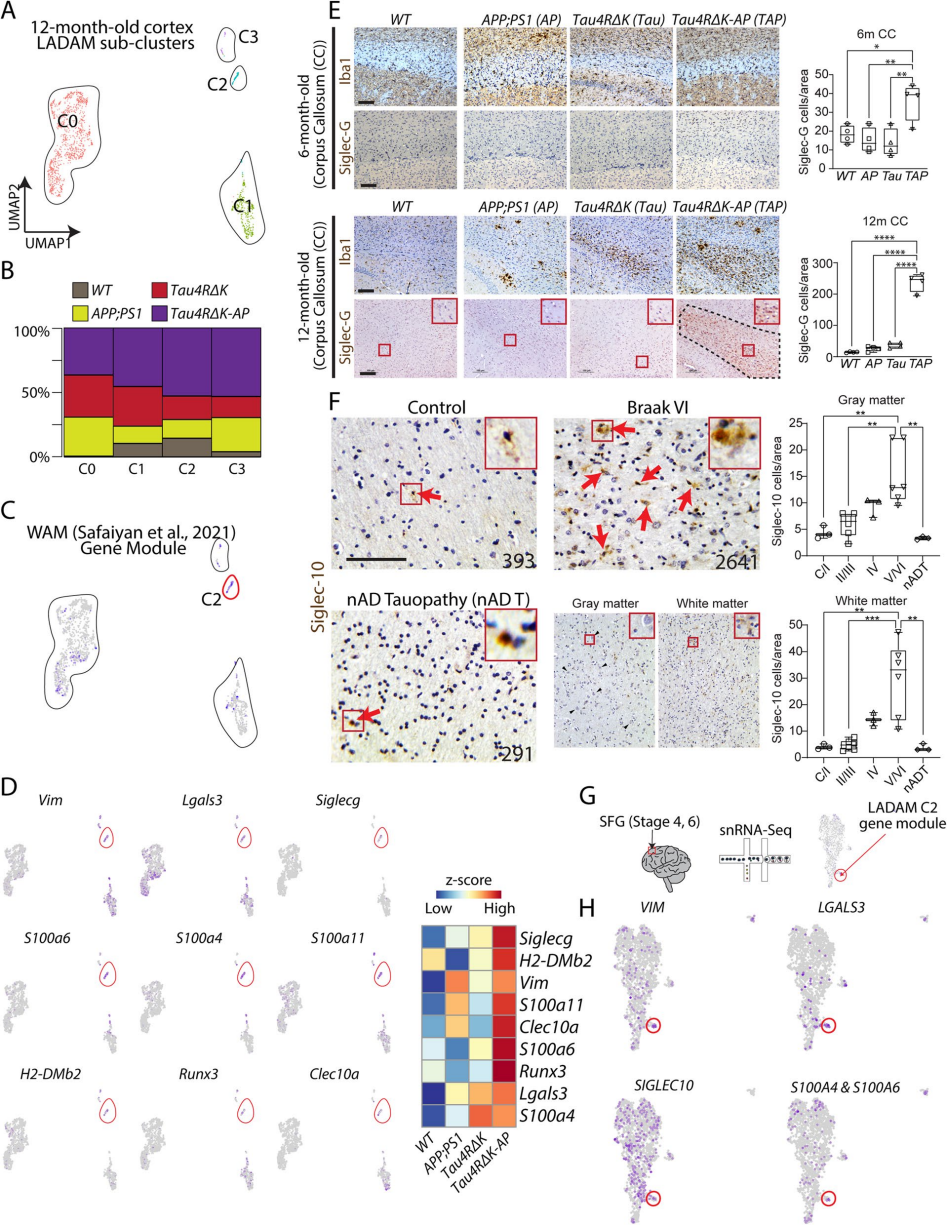
A LADAM subcluster is observed in white-matter-associated microglia. **A** UMAP plot of 12-month-old ‘LADAM’. **B** Bar plot showing the distribution of 4 genotypes across 4 LADAM clusters. **C** UMAP plot of LADAM, showing a small cluster (C2, purple-colored cells) that resemble WAM gene modules [25]. **D** UMAP plots showing gene expressions that are highly enriched genes, including WAM genes (*Vim* and *Lgals3*) in the LADAM C2 cluster (left), and heatmap showing expression of WAM gene modules in C2 across genotypes (right). **E** Iba1 and Siglec-G immunostaining (left) and quantification (right) in 6-month-old (top) and 12-month-old (bottom) corpus callosum in *WT*, *APP;PS1*, *Tau4RΔK*, and *Tau4RΔK-AP*. **F** Siglec-10 immunostaining in human cortex in control, Braak stage 6, and nAD tauopathy (numbers on figure panels indicate BRC# In Table S11, immunostaining of other Braak stages are shown in Fig S15), with bar graphs showing quantification of Siglec-10 positive cells in gray (top) and white (bottom) matter. **G** Schematic showing human snRNA-Seq on Stage 4 and 6 SFG (left) and UMAP plot showing LADAM C2 enriched gene module (right). **H** UMAP plots showing expression of LADAM C2 cluster markers in human snRNA-Seq. Red boxes show high magnification views. CC = Corpus Callosum. Scale bars = 100 μm. * *P* < *0.05*, ** *P* < *0.01*, *** *P* < *0.001*

To further characterize late-stage AD-specific genes, We identified 4 subclusters within the LADAM cluster of AD mouse models (Fig. 6A). Sub-clusters 2 and 3, in particular, were most robustly and selectively expressed in *Tau4RΔK-AP* mice (Fig. 6B, Table S9). Cluster 2, a LADAM sub-cluster that is marked by *Siglecg*, also exhibited genes previously shown to be expressed in White matter-Associated Microglia (WAM), such as *Lgals3*, *Fam20c*, *Vim* (Fig. 6A, C) [25]. In WAM-like LADAM, we observed that this cluster also expressed *S100a6*, *S100a4, S100a11, H2-DMb2, Runx2,* and *Clec10a* (Fig. 6D, Table S9). While a small cluster that resembles WAM was also observed in the 6-month-old mouse cortex (Figure S6J), this small population did not express any LADAM markers in the 12-month-old cortex. Consistent with our scRNA-Seq, we did not detect high-level Siglec-G protein in the 6-month-old corpus callosum, hippocampus, and cortex (Figs. 6E, S14). Siglec-G immunoreactivity was robust in the 12-month-old corpus callosum in *Tau4RΔK-AP* microglia (Figs. 6, S15), as well as in the cortex and hippocampus (Figure S14). However, we failed to observe a strong Siglec-G immunoreactivity in *APP;PS1* or *Tau4RΔK* microglia at the same age, despite increased microglia populations near the corpus callosum (Fig. 6D, S14).

To corroborate this finding in AD, we used antisera directed against Siglec-10 (the human homolog of Siglec-G) [70] to screen AD brains at multiple Braak stages (Table S10). We found Braak stage-dependent accumulation of Siglec-10 in AD brains (Figs. 6F, S15, S16), with Siglec-10 signal significantly increased from Braak stage 4 (Figs. 6F, S16) in both gray and white matters, but a slightly higher number of Siglec-10 + cells were detected in the white matter. However, Siglec-10 signal in non-AD tauopathy is similar to that of healthy controls, consistent with our snRNA-Seq data (Figs. 5G, 6F, S16). To further validate these observations, we checked snRNA-Seq datasets of human postmortem Braak stage 4 and 6 SFG (Fig. 6G, Table S11). We observed a small population of WAM-like LADAM, as seen in 12-month-old *Tau4RΔK-AP* mice (Fig. 6H), expressing genes like *SIGLEC10, S100A6, LGALS3, FAM20C* (Fig. 6H). We have also validated an upregulation of EADAM and LADAM-markers, such as Lgals3 and STAT1 in AD samples (Figure S17).

Siglecs, in particular Siglec10, are AD-enriched, as the level of Siglec-10 + cells in non-AD tauopathy was similar to that of control and low Braak stage AD (Figs. 6F, S16), indicating that Siglec-10 could potentially serve as an AD biomarker and a potential therapeutic target associated with late-stage AD. Some LADAM markers, particularly MHC II genes, were expressed in other neurodegenerative models (Figs. 4, S7), but *S100* family of genes and *Siglecg* was unique to late-stage AD microglia and are seen in both animal models and late-stage AD patient brains. This data further supports the view that induction of the AD-specific LADAM subcluster is Aβ- and tau-dependent.

## Discussion

### Identification of novel disease stage-specific microglial subtypes induced by Aβ plaques and tau pathology

The identification of multiple AD risk alleles implicated in the regulation of inflammation [13, 71] and of DAM induction in response to Aβ plaques [20], strongly supports the hypothesis that microglia-driven neuroinflammation is a key regulator of AD progression. However, critical information regarding microglia subtypes that respond to disease stage-specific canonical AD pathologies of Aβ and tau, particularly during early disease stages, remains elusive. To address this issue, we took advantage of our *Tau4RΔK-AP* mouse model, which exhibits both Aβ plaques and tau deposition, and profiled microglia subtypes during disease progression using scRNA-Seq. To tease apart the influence of Aβ plaques from that of tau deposition, we also profiled microglial subtypes in *APP;PS1* (model of Aβ plaques) and *Tau4RΔK* (model of tau deposition) littermate mice. We identified several disease stage-specific subtypes of microglia that are selectively induced in response to Aβ and/or tau.

We discovered the emergence of a novel microglia subtype, Early-stage AD-Associated Microglia (EADAM), that is selectively induced by both Aβ and tau pathologies but not either Aβ or tau pathologies alone, during early-stage disease before AD-like pathologies are observed. EADAM are characterized by many interferon-pathway-related genes (*Ifit3, Ifit204, Ifit2*) and interferon-regulating transcription factors (*Irf2/7/9)* as well as inflammatory chemokines (*Ccl12, Cxcl10, Ccl5, Ccl2*). Some of the genes in EADAM have been observed in other mouse models of amyloidosis that show early AD-like pathology [48–50], including *APP;PS1* and 5xFAD mouse models. However, given that EADAM-enriched genes are expressed in much higher levels in our *Tau4RΔK-AP* microglia, this strongly supports the view that both Aβ and tau pathologies are required to induce disease stage-specific microglia subtypes in AD.

We also identified previously described DAM in both mouse lines in which Aβ plaques are induced (*APP;PS1* and *Tau4RΔK-AP*) [20, 72]. However, DAM induction in *Tau4RΔK* mice was blunted, supporting the hypothesis that DAM are induced by Aβ plaques and/or inflammation, but not by tau pathology. While Aβ plaques alone are sufficient to induce DAM2, this subtype can be greatly amplified by both Aβ and tau pathologies, giving rise to LADAM. LADAM continues to express classic DAM genes but also upregulates additional genes, including *Cd74* and MHC Class II genes. These broad LADAM marker-expressing microglia (*Cd74* and MHC Class II genes) have been previously shown in some amyloidosis mouse models at late stages (known as activated response microglia) [48–50], but these genes are likely to be contributed by mild tau phosphorylation shown in these amyloidosis mouse models, based on our findings from *Tau4RΔK* (Fig. 2). In addition, since LADAM-like clusters and markers were observed in other neurodegeneration models, such as *TDP43 KO* and *CK-p25* [52, 53], LADAM-enriched genes might be induced by neurodegeneration and/or inflammation in addition to tau pathologies. However, much like EADAM, LADAM-enriched genes were expressed in *Tau4RΔK-AP* microglia, indicating that the full set of LADAM-specific genes is induced by the combination of both Aβ and tau pathologies, and perhaps with neuronal atrophy. Furthermore, the combination of Aβ and tau pathologies induced LADAM to express unique *S100a* genes and *Siglecg*.

In particular, a sub-cluster of LADAM during late-stage disease shares a similar gene signature with previously identified white-matter-associated microglia (WAM). WAM have been previously shown to be present in 24-month-old wild-type mice [25]. Indeed, microglia near white matter tracts were detected in all four genotypes in 12-month-old mice, but only mice with Aβ plaques and tau deposition showed expression of specialized LADAM gene signatures such as *Siglecg.* These data indicated that Aβ plaques and tau deposition induce new molecular signatures in previously identified WAM, leading to the formation of this LADAM subcluster.

While our discovery of novel microglia subtypes has important clinical implications, the mechanisms by which EADAM and LADAM emerge during different stages of disease progression in response to Aβ plaques and tau deposition remain to be fully established. Our current study identifies potential regulators of these microglia populations in response to Aβ plaques and tau deposition. Neuronal loss or brain atrophy induced by aging and/or tau pathologies may also play an essential role in regulating this process.

In summary, our data indicated that while EADAM-related or LADAM-related gene activation represents a general response of microglia to various pathological contexts, there exists a unique gene signature that would define specific microglia subtypes that respond to specific pathology temporally during disease progression. Thus, by elucidating such “microglia response code”, one is expected to discover specific microglia subtypes relevant to the pathological context of human disease. Supporting this view, while we observed both EADAM-like and/or LADAM-like clusters in *TDP43-KO* or *CK-p25* mouse models, there are substantial differences in expression levels of EADAM and LADAM genes in *Tau4R△K-AP* as compared to those of *TDP43-KO* or *CK-p25* as well as *Tau4R△K* and *APP;PS1* mice (Figs. 1, 2, 4, S6). We also found unique LADAM markers in *Tau4R△K-AP* mice that are not expressed in any other neurodegenerative models (Figs. 2, 4, S6). These data strongly support a model in which there exists an EADAM-like and LADAM-like “microglia code” harbored by microglia subtypes that respond to either a general or specific pathological context in the brain. We demonstrate for both Aβ and tau pathologies that this canonical AD pathology context selectively induces novel microglia subtypes in a disease stage-specific manner. These disease stage-dependent “microglial codes” could not only serve as biomarkers for precise diagnosis but also as the therapeutic target for stage-specific intervention of specific neurodegenerative diseases.

### Identifications of disease stage-specific microglia subtypes in AD

The emergence of EADAM during the presymptomatic AD stage and detection of LADAM when AD-like pathologies are visible led to the hypothesis that AD brains may harbor similar unique microglia clusters. EADAM were observed in the entorhinal cortex during early (Braak II) but not late (Braak VI) stage disease, strongly supporting the view that EADAM first emerge in response to initial stages of Aβ plaque formation and tau deposition, corresponding to a stage of negligible or mild cognitive impairment. The microglia cluster expressing an IFN-related gene that is similar to EADAM has been shown in the FTDP-17-linked tau model (P301L) [57] crossed with PS2APP [58], but only when *Trem2* is deficient [55, 56]. Since *Trem2* deficiency can result in the spreading of tau aggregates [73], the FTDP-17-linked tau model (P301L) can result in mild tau aggregation, which resemble the early stages of our mouse model that is characterized by Aβ plaques stimulated pathological conversion of wild-type mouse tau to form tau tangle-like aggregates. These previous finding [56] aligns well with our finding that Aβ plaques and tau deposition are required to give rise to EADAM.

In contrast, the emergence of LADAM in both the entorhinal cortex and superior frontal gyrus did not occur until the Braak IV stage, implying that these subtypes are induced in response to more severe Aβ and tau pathologies accompanied by neuronal loss during late-stage disease. Some LADAM-enriched genes such as *HLA-DRB1* and *HLA-DRB5* positively correlate with AD pathology [74] and share a few similar molecular signatures (e.g. MHC Class II genes) with lipid droplet-accumulating microglia [75]. We also observed an upregulation of *S100A* genes in late-stage AD.

Although interferon-signaling-related genes (EADAM-enriched) and MHC and S100 family genes (LADAM-enriched) are observed during human early- and late-AD stages, respectively, not all EADAM- or LADAM-enriched genes were detected in human samples. We were also unable to identify well-defined and distinct microglial clusters in human AD snRNA-Seq, in agreement with other studies. This might reflect the much longer time course of AD progression in humans compared to mouse models, potentially leading to less well-defined microglia subtypes. The cluster-basis analysis method which is typically used for the analysis of cell states in animal models might not be the best method to define microglia subtypes in human disease states. Nonetheless, gene profiling studies conducted in appropriate animal models of AD could yield insights to facilitate the profiling of corresponding unique microglia subtypes or specific genes in AD brains, a task that can be technically challenging to interpret.

The fact that EADAM are first detected in 6-month-old *Tau4RΔK-AP* mice and Braak stage II AD datasets, while LADAM are first detected in 12-month-old *Tau4RΔK-AP* mice and Braak stage IV AD datasets, demonstrates that disease stage-dependent changes in microglial subtypes in mice with Aβ and tau pathologies mimic those seen in AD. *Tau4RΔK-AP* mice faithfully model not only the development of canonical AD pathologies, including the pathological conversion of wild-type mouse tau [24], but also the disease stage-specific emergence of EADAM and LADAM in response to both Aβ plaques and tau deposition, emphasizing that our mouse model will prove useful in both analyzing AD disease mechanisms and testing therapeutic strategies for treating AD.

We currently do not know the functions of these microglia subtypes, but pathway analysis (Fig. 1) of EADAM shows enrichment of ‘Response to interferon-beta. EADAM is present in *Tau4RΔK-AP* mice before AD-like pathology appears, and our current hypothesis is that EADAM at 6-month-old could potentially provide a protective role [76, 77]. As for LADAM, our pathway analysis (Fig. 2) shows that ‘Antigen presentation via MHC Class II’ and ‘Antigen processing and presentation’ are strongly associated with LADAM, which are common pathways associated with neurodegeneration [78]. LADAM might be involved in neuronal degeneration, as MHC Class II has been shown to be important in neuronal degeneration by microglia in some neurodegeneration [79].

### Siglec signaling in EADAM and LADAM

The identification of disease stage-enriched EADAM and LADAM in our *Tau4RΔK-AP* model and AD brains raise the possibility that specific signaling pathways may be important in the emergence of these microglial subtypes. CD33 (also known as Siglec-3) [80], is one of the several microglial-specific genes that have been linked to AD susceptibility via genome-wide association studies (GWAS) [81] and both mediate immune suppression by binding sialoglycan targets and attenuates phagocytosis by microglia during AD progression [80, 82]. As a result, we first focused on characterizing the expression of sialic acid-binding immunoglobulin-like lectin (Siglec) family of proteins. Human Siglecs belongs to a family of 14 distinct transmembrane proteins, many of which are expressed in overlapping subsets of immune cells and inhibit immune activation [70, 83]. In addition to Siglec-3, other members of the Siglec family might regulate microglial function in AD. Our findings showing specific enrichment of Siglec-pathway genes in EADAM and LADAM strongly support this hypothesis. Siglec-G is enriched in microglia near myelinated regions, whereas Siglec-F + microglia are more broadly expressed throughout the brain. Our data suggest that Siglec-F is dependent on Aβ deposition, that Siglec-G is dependent on tau deposition, and that Aβ and tau deposition synergistically enhance the expression of both Siglecs. The observation that *Siglecg* is highly expressed in LADAM while Siglec-10 (the human homolog of Siglec-G), is associated with late-stage AD pathologies exhibiting robust Aβ and Tau deposition, further supports the view that Siglec10 signaling underlies the activation of LADAM in AD. Moreover, EADAM selectively expressed both *Siglec1* and *Siglecf*, while DAM and MHC Class II clusters both expressed *Siglecg*, indicating that Siglec-1, Siglec-F, and Siglec-G, respectively, represent potential biomarkers for the detection of early- and late-stage disease. Based on these findings, we hypothesize that Siglec-regulated signaling pathways may have an important function in regulating disease stage-specific microglia during AD progression. Future studies will directly address disease-associated functions of Siglec-regulated signaling pathways.

However, it is often difficult to identify Siglec orthologues between mice and humans, as this gene family evolved rapidly [83, 84]. Mice do not express Siglec-8, and mouse Siglec-3 (mSiglec‑3) has different effector domains, binding specificity, cellular distribution, and biological properties compared to those of human Siglec-3 (hSiglec‑3) [85, 86]. However, current studies suggested there is functional conservation between mice and humans in Siglec gene family. Siglec In mouse microglia, immune inhibitory Siglec-F is among the most highly up-regulated genes (27-fold) in experimental neurodegenerative proteinopathy [87]. Siglec-F, a likely functional orthologue of Siglec-8 [88], is expressed in eosinophils as well as microglia [87, 88], binds to the same sialoglycans and sialoglycan mimetics as Siglec-8 (unlike mSiglec-3) [89, 90]. Both Siglec-F and Siglec-8 also regulate eosinophilic inflammation [91, 92]. Siglec-F was upregulated on a subset of reactive microglia in models of neurodegeneration, indicating the important role of Siglec-F/Siglec-8 in regulating microglial activation during neurodegeneration [93]. In a recent study, RPTPζ^S3L^, a sialoglycoprotein was identified as a specific ligand in the human cerebral cortex that binds CD33 as well as Siglec-8 [94]. Interestingly, RPTPζ isoform in mice binds mouse Siglec-F and cross-reacts with human CD33 and Siglec-8 [94], suggesting Siglec-F shares a similar ligand with CD33 and Siglec8 and is a potential functional counterpart of CD33 in humans. Importantly, we observed similar expression patterns of siglec signaling between mice and humans, such as Siglec-F/8 expression emerged at earlier disease stages than Siglec-G/10 in both our mouse models and in AD, indicating that our mouse model could serve as an important tool to study the functions of Siglec signaling in AD pathological stages and microglia subtypes.

## Conclusions

In this study, microglia subtypes in a mouse model of AD that develop both Aβ and tau pathologies were profiled using scRNA-Seq. We found that during early-stage disease in 6-month-old *Tau4RΔK-AP*, the presence of both Aβ and tau pathologies induced a novel microglia subtype EADAM, which is distinct from DAM and express multiple interferon-regulated genes. In late-stage disease (12-month-old *Tau4RΔK-AP* mice), we found that another novel microglia subtype LADAM emerged in response to tau pathology, which expresses MHC and S100 family genes. We further observed a unique subtype of LADAM located near white matter, which is molecularly similar to a previously identified white matter-associated microglial subtype (WAM). We found that the signature genes in EADAM were also associated with a subgroup of microglia in the brains of early (Braak II) stages of AD. While LADAM microglia, including WAM-like LADAM, were observed in the late (Braak VI), but not early (Braak II) stages of AD. Corroborating these findings, we found that *Siglec* family members are associated with specific subsets of microglia that are activated in a disease-stage-specific manner in both mouse models of AD and AD patients. For example, Siglec-F and CD22 are upregulated in response to Aβ pathology and is selectively expressed in Aβ-associated DAMs, while Siglec-G/10 is upregulated in white-matter-associated LADAM in late-stage AD. These findings are consistent with a model whereby Aβ or tau pathologies stimulate the activation of microglia in different patterns, both Aβ and tau deposition are required to mediate the disease stage-specific induction of EADAM and LADAM, and can alter Siglec signaling across AD pathological stage and microglia subtypes. These findings may have important implications for the identification of novel molecular targets, and offer new targets for the development of pre-symptomatic biomarkers and therapeutic strategies for AD.

Our data indicated that while EADAM-related or LADAM-related gene activation represents a general response of microglia to various pathological contexts, there exists a unique gene signature that would define specific microglia subtypes that respond to specific pathology temporally during disease progression. Thus, by elucidating such “microglia response code”, one is expected to discover specific microglia subtypes relevant to the pathological context in human disease. Supporting this view, while we observed both EADAM-like and/or LADAM-like clusters in *TDP43-KO* or *CK-p25* mouse models, there are substantial differences in expression levels of EADAM and LADAM genes in *Tau4R△K-AP* as compared to those of *TDP43-KO* or *CK-p25* as well as *Tau4R△K* and *APP;PS1* mice. We also found unique LADAM markers in *Tau4R△K-AP* mice that are not expressed in any other neurodegenerative models. These data strongly support a model in which there exists an EADAM-like and LADAM-like “microglia code” harbored by microglia subtypes that respond to either a general or specific pathological context in the brain. We demonstrate for both Aβ and tau pathologies that this canonical AD pathology context selectively induces novel microglia subtypes in a disease stage-specific manner. These disease stage-dependent “microglial codes” could not only serve as biomarkers for precise diagnosis but also as the therapeutic target for stage-specific intervention of specific neurodegenerative diseases.

Lastly, similar microglia cell types that share partial overlap in gene expression and low levels of expression of EADAM and LADAM markers can be detected in other mouse models of neurodegenerative disorders. While we have given unique names to AD-associated microglia in our AD models, a more standardized nomenclature for disease-associated microglial subtypes needs to be worked out by the research community in the near future.

## Supplementary Information

**Additional file 1: Figure S1.** Histological validation of tau and Aβ pathologies in 6-month-old and 12-month-old *Tau4RΔK-AP *mice. A-H, Immunostaining of Aβ (6E10) (A-D), tau (pS422) (E-H) at 6-month-old (A, B, E, F,) and 12-month-old (C, D, G, H); in the Cortex (A, C, E, G), and Hippocampus (B, D, F, H), in *WT*, *APP;PS1*, *Tau4RΔK*, *Tau4RΔK-AP*. I, Quantification of NeuN-positive cells in the hippocampus at 6-month-old (left) and 12-month-old (right). J, Quantification of brain weight at 12-month-old. K, Quantification of Aβ deposition covered areas in the hippocampus of 12-month-old mice. L-N, Immunostaining of Thioflavin T (ThioT) at 12-month-old; in the Cortex (L), and Hippocampus (M), in *APP;PS1*, *Tau4RΔK-AP* mice. Quantification of ThioT/Plaques are shown in N. O-Q, Quantification of plaque numbers (O), plaque area (P), and plaque sizes (Q), and in the 6-month-old and 12-month-old *Tau4RΔK-AP* mice. Ctx = Cortex, Hippo = Hippocampus. Scale bars = 100 μm. NS = not significant, * *P < 0.05*, ** *P <0.01*, *** P < 0.001, **** *P < 0.0001*.

**Additional file 2: Figure S2. **Histological validation of microglia distribution in 6-month-old and 12-month-old *Tau4RΔK-AP *mice. A-D, Immunostaining of IBA1 at 6-month-old (A, B) and 12-month-old (C, D); in the Cortex (A, C), and Hippocampus (B, D), in *WT*, *APP;PS1*, *Tau4RΔK*, *Tau4RΔK-AP*. E-G, Quantification of IBA1 staining in the cortex (E), hippocampus (F), and corpus callosum (G) at 6-month-old and 12-month-old. Ctx = Cortex, Hippo = Hippocampus, CC = Corpus Callosum. Scale bars = 100 μm. NS = not significant, * *P < 0.05*, ** *P <0.01*, *** P < 0.001, **** *P < 0.0001*.

**Additional file 3: Figure S3. **Distribution of cell types in 6-month-old cortex across genotypes. A, Schematic design of experiment: 4 genotypes (*WT, APP;PS1, Tau4RΔK, Tau4RΔK-AP*) in the cerebral cortex at 6-month-old and 12-month-old. B, Distribution of cell types across genotypes in the 6-month-old cortex. Numbers indicate the number of captured cell types. C, UMAP plot showing captured cell types in the 6-month-old cortex (all genotypes). D, UMAP plot showing the density of the captured cell types across genotypes in the 6-month-old cortex. E, UMAP plot showing DAM marker gene *Cst7*. F, UMAP plot showing DAM marker gene *Gpnmb*. BAM = brain-associated macrophages, Ctx = Cortex, MSC = muscle stem cells, OPC = oligodendrocyte precursor cells, VSMC = vascular smooth muscle cells. N = 3/genotype, *WT* = 56668 cells total*, APP;PS1* = 52797 cells total*, Tau4RΔK* = 49008 cells total*, Tau4RΔK-AP* = 55306 cells total.

**Additional file 4: Figure S4. **Distribution of cell types in 12-month-old cortex across genotypes. A, Schematic design of experiment: 4 genotypes (*WT, APP;PS1, Tau4RΔK, Tau4RΔK-AP*) in the cerebral cortex at 6 and 12-month-old. B, Distribution of cell types across genotypes in 12-month-old cortex. Numbers indicate the number of captured cell types. C, UMAP plot showing captured cell types in the 12-month-old cortex (all genotypes). D, UMAP plot showing the density of the captured cell types for each genotype in the 12-month-old cortex. E, UMAP plot showing DAM marker gene *Cst7*. F, UMAP plot showing DAM marker gene *Gpnmb*. BAM = brain-associated macrophages, Ctx = cortex, MSC = muscle stem cells, OPC = oligodendrocyte precursor cells, VSMC = vascular smooth muscle cells. *N* = 3/genotype, *WT* = 47455 cells total*, APP;PS1* = 42247 cells total*, Tau4RΔK* = 41152 cells total*, Tau4RΔK-AP *= 35431 cells total.

**Additional file 5: Figure S5. **Distribution of clusters across genotypes. A, Distribution of cell types across 6-month-old scRNA-Seq triplicates. B, Distribution of cell types across 12-month-old scRNA-Seq triplicates. BAM = brain-associated macrophages, MSC = muscle stem cells, OPC = oligodendrocyte precursor cells, VSMC = vascular smooth muscle cells. R1 = Biological replicate 1, R2 = Biological replicate 2, R3 = Biological replicate 3.

**Additional file 6: Figure S6. **Analysis of NF-kB pathway genes in 6-month-old AD mouse models. Heatmap plots showing expressions of NF-kB pathway genes at 6-month-old across microglia subtypes (A), and genotypes (B).

**Additional file 7: Figure S7.** Analysis of EADAM and LADAM markers in other mouse models of neurodegenerative disease and AD samples. A, UMAP plot showing *CK-p25* neurodegenerative scRNA-Seq microglia dataset from Mathys et al., 2017. B, UMAP plots showing EADAM and LADAM-enriched genes. Note that while IFN and MHC genes are present, S100 genes and *Siglecg* are absent in the dataset.

**Additional file 8: Figure S8. **Analysis of distinct expression profiles that are associated with AD pathology in AD mouse models. Heatmap plots showing homolog expressions of Aβ-associated AD1-enriched (A), and Tau-associated AD2-enriched (B) genes from Gerrits et al., 2021 human snRNA-Seq dataset [59], in 6-month-old 12-month-old, in *WT*, *APP;PS1*, *Tau4RΔK*, *Tau4RΔK-AP*.

**Additional file 9: Figure S9.** Histological validation of DAM markers in AD mouse models. A-C, Immunostaining of LPL at 6-month-old (A) and 12-month-old (B, C); in the Cortex (B), and Hippocampus (A, C), in *WT*, *APP;PS1*, *Tau4RΔK*, *Tau4RΔK-AP*. Black arrowheads indicate examples of LPL-positive cells. D-E, Immunostaining of IBA1 (Red) and LPL (Green) at 6-month-old in the cortex in *APP;PS1 *(D), *Tau4RΔK-AP* (E). White arrowheads indicate IBA1 and LPL double-positive cells. F-G, Immunostaining of CD22 (Siglec-2) at 12-month-old; in the cortex (F), and hippocampus (G), in *WT*, *APP;PS1*, *Tau4RΔK*, *Tau4RΔK-AP*. Black arrowheads indicate examples of LPL-positive cells. Ctx = Cortex, Hippo = Hippocampus. Scale bars = 50 μm.

**Additional file 10: Figure S10.** Histological validation of DAM/LADAM marker in AD mouse models. A-D, Immunostaining of Lgals3 at 6-month-old (A) and 12-month-old (B-D); in the cortex (A, B), hippocampus (C), and corpus callosum (D), in *WT*, *APP;PS1*, *Tau4RΔK*, *Tau4RΔK-AP*. Black arrowheads indicate examples of Lgals3-positive cells. E-G, Quantification of Lgals3 staining in the cortex (E), hippocampus (F), and corpus callosum (G) at 12-month-old, in *WT*, *APP;PS1*, *Tau4RΔK*, *Tau4RΔK-AP*. Ctx = Cortex, Hippo = Hippocampus, CC = Corpus Callosum. * *P < 0.05*, ** *P <0.01*, *** P < 0.001, **** *P < 0.0001*.

**Additional file 11: Figure S11.** Histological validation of LADAM marker in AD mouse models. A-C, Immunostaining of Tau(pS422) (Red) and Lgals3 (Green) at 12-month-old in the cortex (A), hippocampus (B), and corpus callosum (C) in *Tau4RΔK*. D, Immunostaining of GFAP (Red) and Lgals3 (Green) at 12-month-old in the corpus callosum in *Tau4RΔK*. E-F, Immunostaining of IBA1 (Red) and Lgals3 (Green) at 12-month-old in the corpus callosum in *Tau4RΔK* (E), *Tau4RΔK-AP *(F). Ctx = Cortex, Hippo = Hippocampus, CC = Corpus Callosum. Scale bars = 50 μm.

**Additional file 12: Figure S12.** Histological validation of EADAM/LADAM markers in AD mouse models. A-B, Immunostaining of STAT1 (Red) and GFAP (Green) at 12-month-old in *APP;PS1* (A), *Tau4RΔK-AP *(B). C-F, Immunostaining of IBA1 (Red) and STAT1 (Green) at 6-month-old (E) and 12-month-old (C, D, F) in *APP;PS1* (C), *Tau4RΔK* (D) *Tau4RΔK-AP *(E, F). Scale bars = 50 μm.

**Additional file 13: Figure S13.** Analysis of EADAM and LADAM markers in AD samples. Heatmap plots showing homolog expression of EADAM (A), and LADAM (B) genes in the human snRNA-Seq dataset.

**Additional file 14: Figure S14.** Siglec-genes show cluster- and genotype-specific expression patterns, and histological validation of Siglec-F and Siglec-G across genotypes in 6 and 12-month-old mice. A, UMAP plot showing microglial clusters in the 6 and 12-month-old cortex (all genotypes). B, Heatmap plot showing Siglec genes expressions across microglial clusters. C, D, UMAP plot showing *Siglecf* expression in *WT*, *APP;PS1*, *Tau4RΔK*, *Tau4RΔK-AP* at 6-month-old (C) and 12-month-old (D) (top). Note a higher expression of *Siglecf *in DAM2 in *APP;PS1* and *Tau4RΔK-AP* mice at 12-month-old. Siglec-F immunostaining was performed in the cortex in *WT*, *Tau4RΔK*, *APP;PS1*, *Tau4RΔK-AP* at 6-month-old (C) and 12-month-old (D) (bottom). E-I, Immunostaining of Siglec-F (E, F), and Siglec-G (G-I), in *WT*, *APP;PS1*, *Tau4RΔK*, *Tau4RΔK-AP*; at 6-month-old (E, G) and 12-month-old (F, H, I); hippocampus (E, F, H), and the cortex (F, G, I). J, UMAP plot of 6-month-old microglia, showing a small cluster (highlighted in red) that resembles molecular genes of WAM [25]. K, Quantification of Siglec-G staining in the 6-month-old and 12-month-old cortex and hippocampus. Ctx = Cortex, Hippo = Hippocampus. Red boxes in C, D, E, F, I show high magnification views. Scale bars = 100 μm. * *P < 0.05*, ** *P <0.01*, *** P < 0.001, **** *P < 0.0001*.

**Additional file 15: Figure S15.** Histological validation of Siglec-G/Siglec-10 expression in microglia in AD mouse models and human postmortem samples. A-B, Immunostaining of IBA1 (Red) and Siglec-G (Green) at 12-month-old in the CC in *Tau4RΔK* (A), *Tau4RΔK-AP *(B). C, Immunostaining of IBA1 (Green) and Siglec-10 (Red) at Braak stage VI. CC = Corpus Callosum. Scale bars = 50 μm.

**Additional file 16: Figure S16.** Histological validation of tau and Aβ pathologies and Siglec-10 during AD progression and in nAD Tauopathy. Immunostaining of Tau (pS422) (A), Aβ (6E10) (B), and Siglec-10 (C) in Control, Braak stage 2, Braak stage 3, Braak stage 4, Braak stage 5, Braak stage 6, and in nAD Tauopathy in the cortex. Numbers indicate BRC# in Table S10. Red boxes show high magnification views. Scale bars = 100 μm.

**Additional file 17: Figure S17.** Histological validation of markers in AD pathology. Immunostaining of IBA1 (Red) and LPL (A, Green); Lgals3 (B, Green); STAT1 (C-D, Green) in Control (D) and AD (A-C). White arrows indicate examples of double-positive cells. Scale bars = 50 μm.

**Additional file 18: Table S1.** Differential gene expression as measured by scRNA-Seq in 6-month-old cortex across cell types. **Table S2.** Differential gene expression as measured by scRNA-Seq in 12-month-old cortex across cell types. **Table S3.** Differential gene expression as measured by scRNA-Seq in 6-month-old microglia subtypes. **Table S4.** Differential gene expression as measured by scRNA-Seq in 6-month-old microglia across genotypes. **Table S5.** Differential gene expression as measured by scRNA-Seq in 12-month-old microglia subtypes. **Table S6.** Differential gene expression as measured by scRNA-Seq in 12-month-old microglia across genotypes. **Table S7.** Differential gene expression as measured by scRNA-Seq in TDP43 KO^6->9^ microglia subtypes. **Table S8.** Differential gene expression as measured by scRNA-Seq in TDP43 KO^12->15^ microglia subtypes. **Table S9.** Differential gene expression as measured by scRNA-Seq in 12-month-old LADAM subtypes. **Table S10.** Characteristics of postmortem brains used in this study for histological analysis. **Table S11. **Characteristics of postmortem brains used in this study for snRNA-Seq.

## Data Availability

Further information and requests for resources and reagents should be directed to and will be fulfilled by the Lead Contact, Tong Li (tli1@jhmi.edu). All unique/stable reagents generated in this study are available from the Lead Contact without restriction. Single-cell RNA-seq data have been deposited at GEO (GSE175546) and are publicly available as of the date of publication. Accession numbers are listed in the key resources table. All other data reported in this paper will be shared by the lead contact upon request.

## Abbreviations

SFG: Superior frontal gyrus
ERC: Entorhinal cortex
EADAM: Early-stage AD-Associated Microglia
LADAM: Late-stage AD-Associated Microglia
AD: Alzheimer’s disease
Aβ: Amyloid-β
scRNA-Seq: Single-cell RNA-sequencing
Siglec: Sialic acid-binding immunoglobulin-type lectin
FFPE: Formalin-fixed paraffin-embedded
H&E: Hematoxylin and eosin
APP;PS1: APP^swe^; PS1ΔE9
PART: Primary-age-related-tauopathy
ADRC: Johns Hopkins Alzheimer’s Disease Research Center
ThioT: Thioflavin-T
