# Supplementary figures and images for: Amyloid-beta and tau pathologies act synergistically to induce novel disease stage-specific microglia subtypes
Source: Mol Neurodegener. 2022 Dec 17;17:83. doi: 10.1186/s13024-022-00589-x (PMC9762062; doi:10.1186/s13024-022-00589-x)

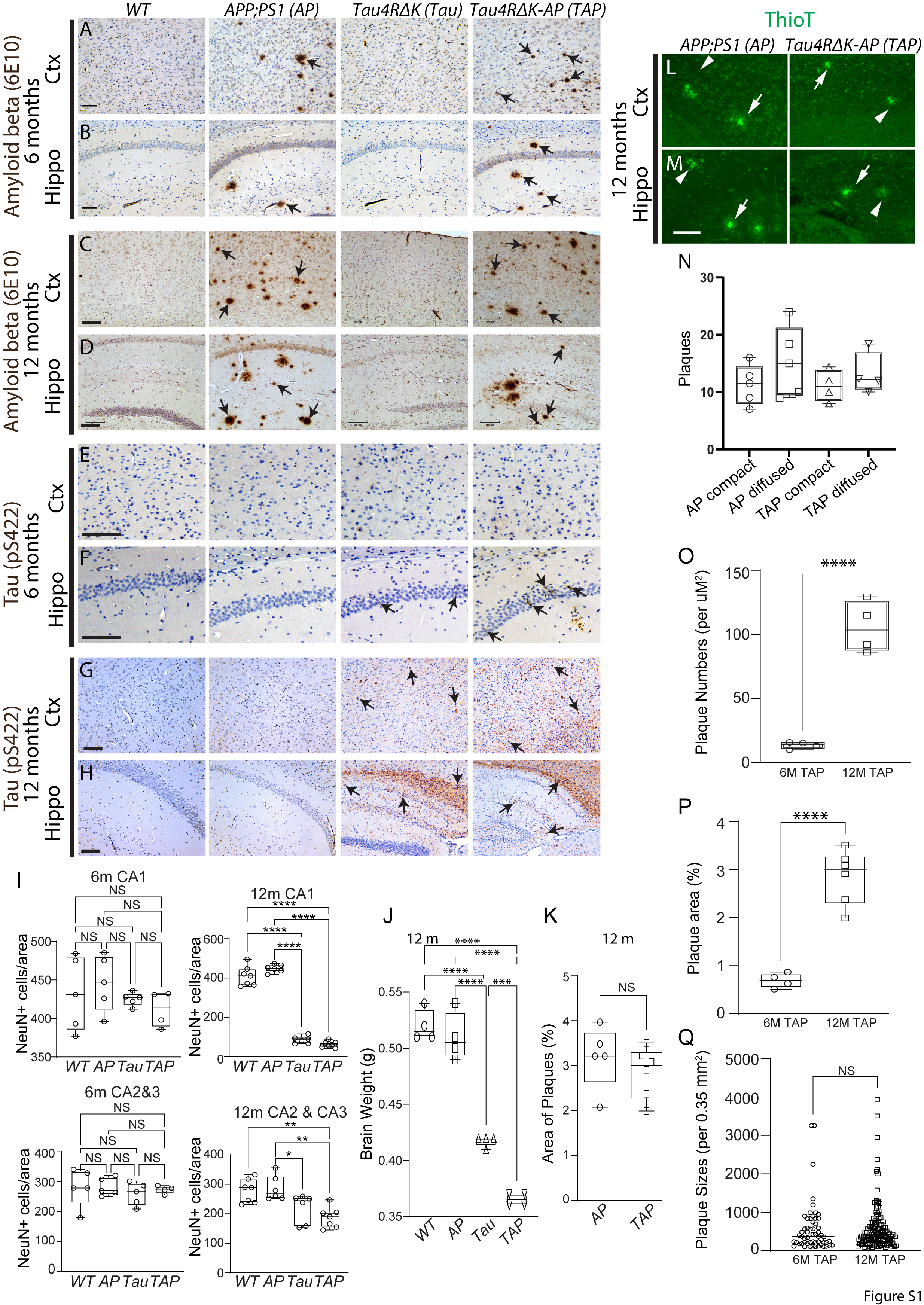

Supplement: Supplementary file 1 — Additional file 1: Figure S1. Histological validation of tau and Aβ pathologies in 6-month-old and 12-month-old Tau4RΔK-AP mice. A-H, Immunostaining of Aβ (6E10) (A-D), tau (pS422) (E-H) at 6-month-old (A, B, E, F,) and 12-month-old (C, D, G, H); in the Cortex (A, C, E, G), and Hippocampus (B, D, F, H), in WT, APP;PS1, Tau4RΔK, Tau4RΔK-AP. I, Quantification of NeuN-positive cells in the hippocampus at 6-month-old (left) and 12-month-old (right). J, Quantification of brain weight at 12-month-old. K, Quantification of Aβ deposition covered areas in the hippocampus of 12-month-old mice. L-N, Immunostaining of Thioflavin T (ThioT) at 12-month-old; in the Cortex (L), and Hippocampus (M), in APP;PS1, Tau4RΔK-AP mice. Quantification of ThioT/Plaques are shown in N. O-Q, Quantification of plaque numbers (O), plaque area (P), and plaque sizes (Q), and in the 6-month-old and 12-month-old Tau4RΔK-AP mice. Ctx = Cortex, Hippo = Hippocampus. Scale bars = 100 μm. NS = not significant, * P < 0.05, ** P <0.01, *** P < 0.001, **** P < 0.0001. [file 13024_2022_589_MOESM1_ESM.tif]

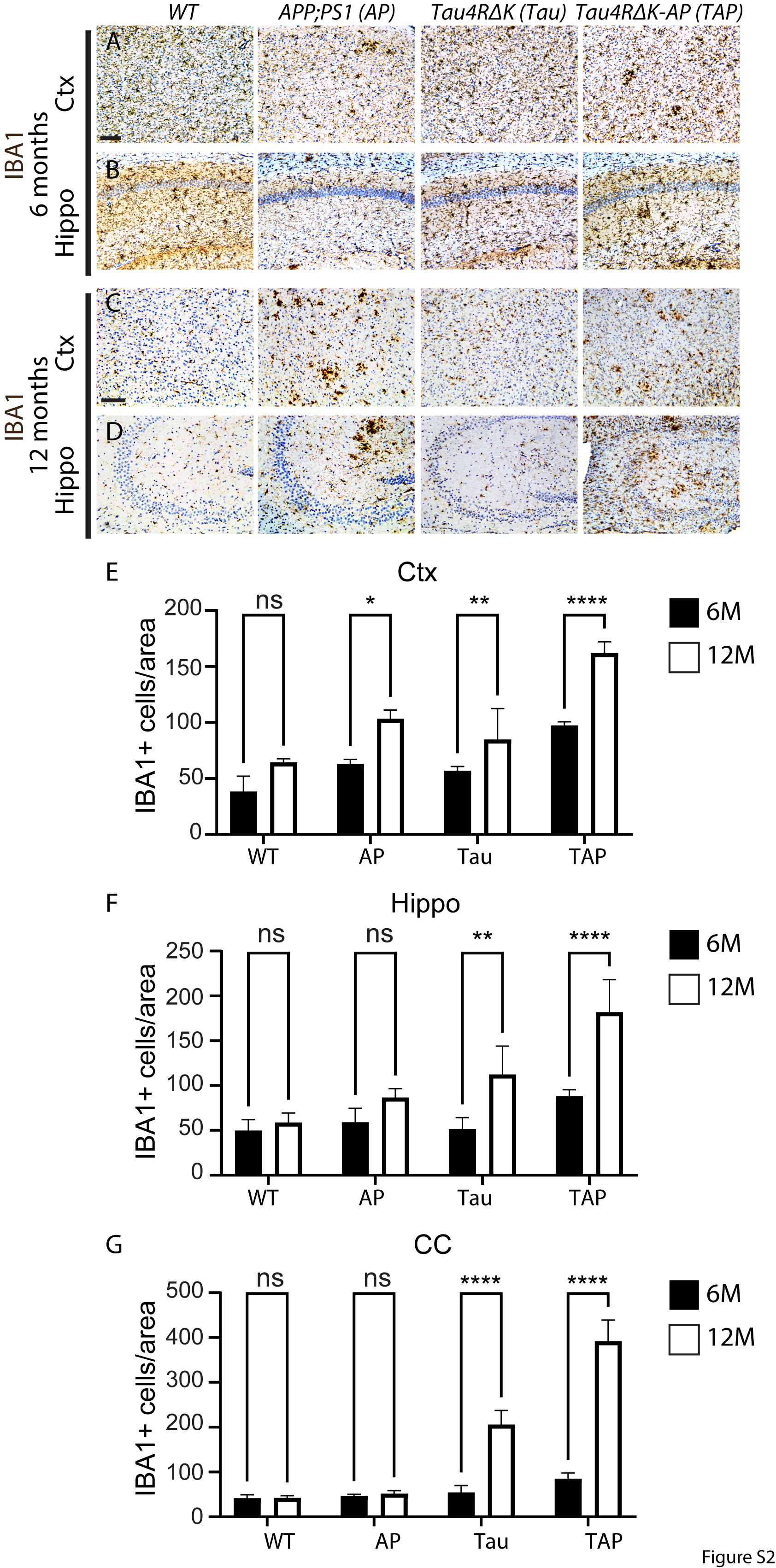

Supplement: Supplementary file 2 — Additional file 2: Figure S2. Histological validation of microglia distribution in 6-month-old and 12-month-old Tau4RΔK-AP mice. A-D, Immunostaining of IBA1 at 6-month-old (A, B) and 12-month-old (C, D); in the Cortex (A, C), and Hippocampus (B, D), in WT, APP;PS1, Tau4RΔK, Tau4RΔK-AP. E-G, Quantification of IBA1 staining in the cortex (E), hippocampus (F), and corpus callosum (G) at 6-month-old and 12-month-old. Ctx = Cortex, Hippo = Hippocampus, CC = Corpus Callosum. Scale bars = 100 μm. NS = not significant, * P < 0.05, ** P <0.01, *** P < 0.001, **** P < 0.0001. [file 13024_2022_589_MOESM2_ESM.tif]

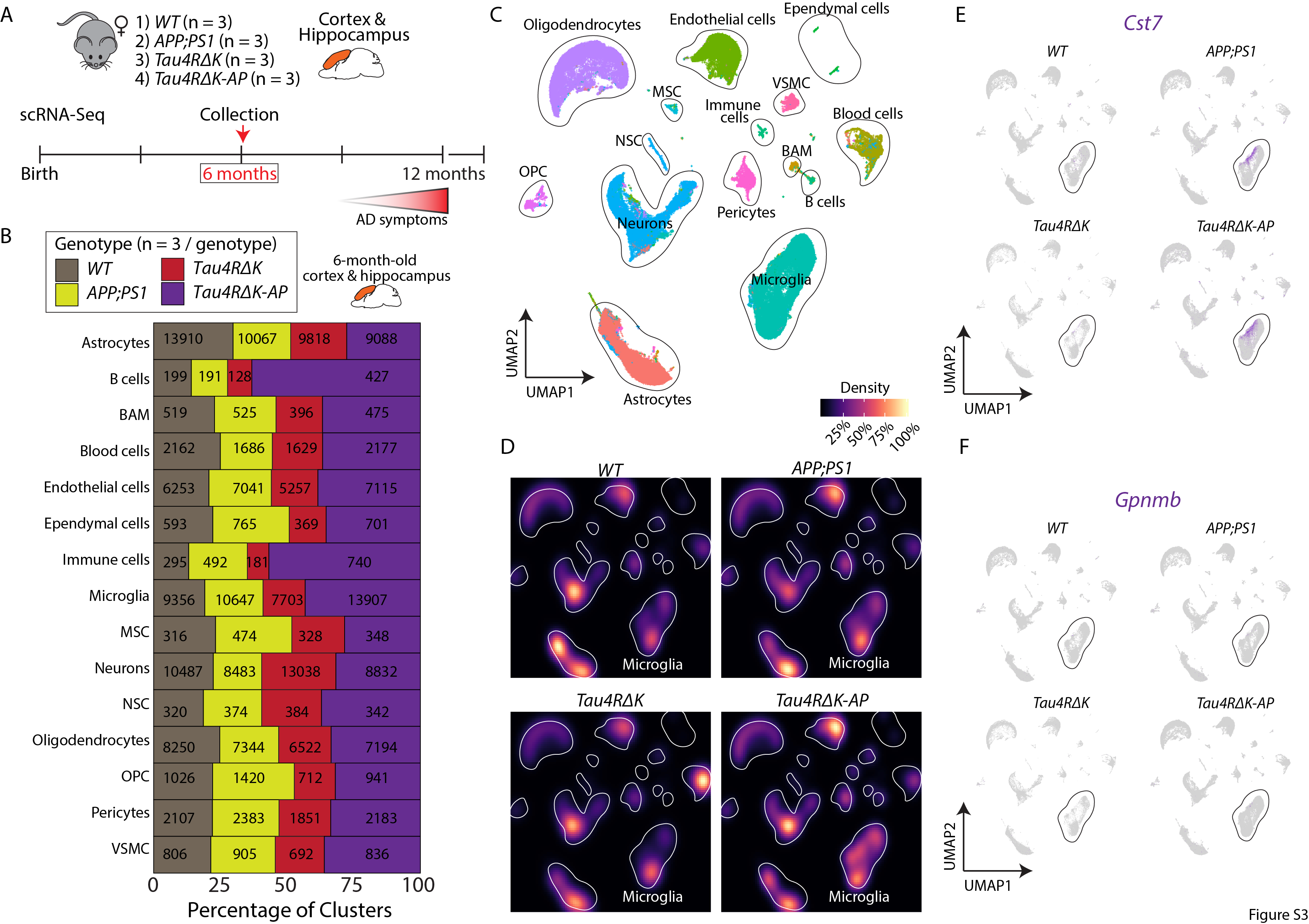

Supplement: Supplementary file 3 — Additional file 3: Figure S3. Distribution of cell types in 6-month-old cortex across genotypes. A, Schematic design of experiment: 4 genotypes (WT, APP;PS1, Tau4RΔK, Tau4RΔK-AP) in the cerebral cortex at 6-month-old and 12-month-old. B, Distribution of cell types across genotypes in the 6-month-old cortex. Numbers indicate the number of captured cell types. C, UMAP plot showing captured cell types in the 6-month-old cortex (all genotypes). D, UMAP plot showing the density of the captured cell types across genotypes in the 6-month-old cortex. E, UMAP plot showing DAM marker gene Cst7. F, UMAP plot showing DAM marker gene Gpnmb. BAM = brain-associated macrophages, Ctx = Cortex, MSC = muscle stem cells, OPC = oligodendrocyte precursor cells, VSMC = vascular smooth muscle cells. N = 3/genotype, WT = 56668 cells total, APP;PS1 = 52797 cells total, Tau4RΔK = 49008 cells total, Tau4RΔK-AP = 55306 cells total. [file 13024_2022_589_MOESM3_ESM.png]

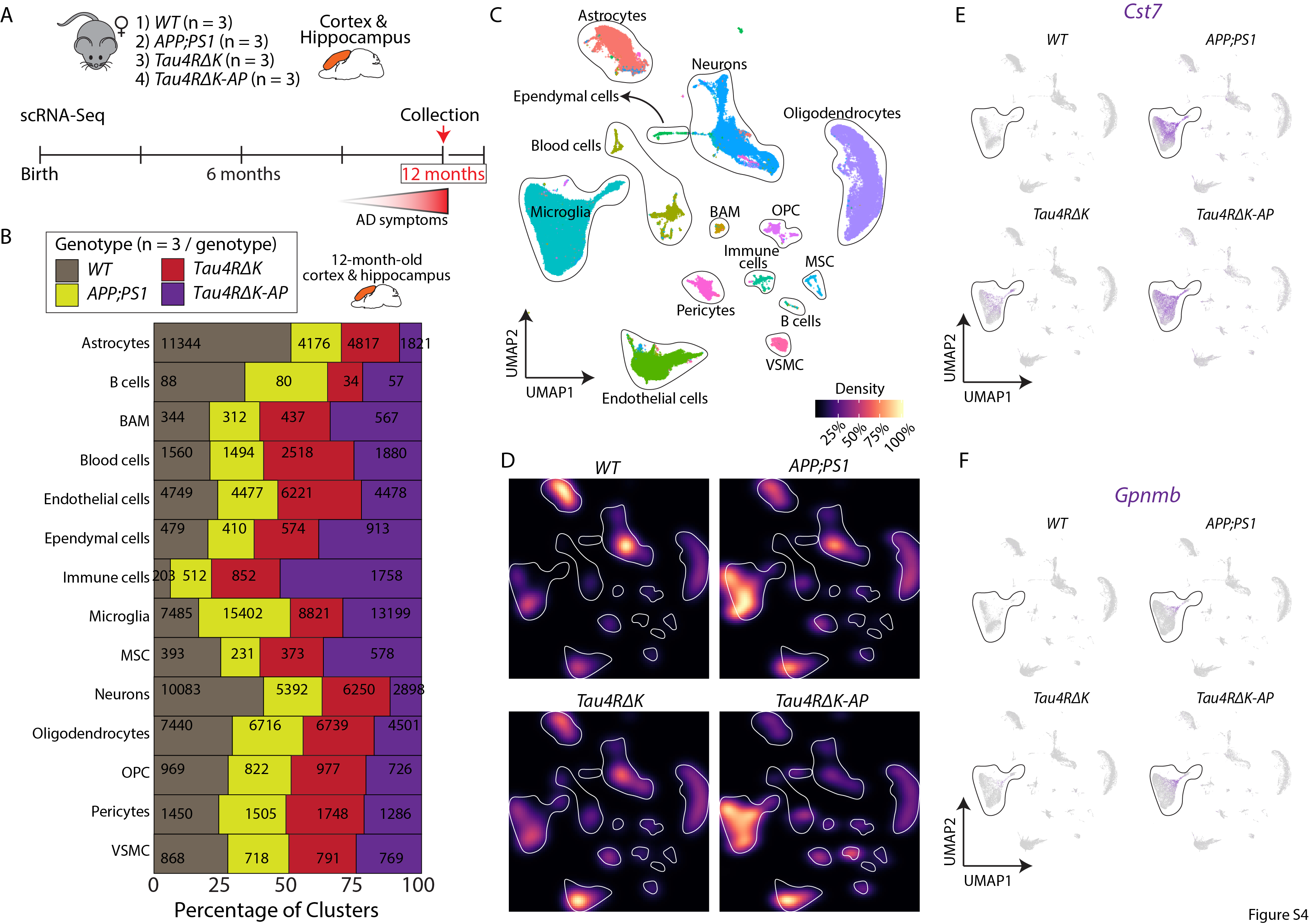

Supplement: Supplementary file 4 — Additional file 4: Figure S4. Distribution of cell types in 12-month-old cortex across genotypes. A, Schematic design of experiment: 4 genotypes (WT, APP;PS1, Tau4RΔK, Tau4RΔK-AP) in the cerebral cortex at 6 and 12-month-old. B, Distribution of cell types across genotypes in 12-month-old cortex. Numbers indicate the number of captured cell types. C, UMAP plot showing captured cell types in the 12-month-old cortex (all genotypes). D, UMAP plot showing the density of the captured cell types for each genotype in the 12-month-old cortex. E, UMAP plot showing DAM marker gene Cst7. F, UMAP plot showing DAM marker gene Gpnmb. BAM = brain-associated macrophages, Ctx = cortex, MSC = muscle stem cells, OPC = oligodendrocyte precursor cells, VSMC = vascular smooth muscle cells. N = 3/genotype, WT = 47455 cells total, APP;PS1 = 42247 cells total, Tau4RΔK = 41152 cells total, Tau4RΔK-AP = 35431 cells total. [file 13024_2022_589_MOESM4_ESM.png]

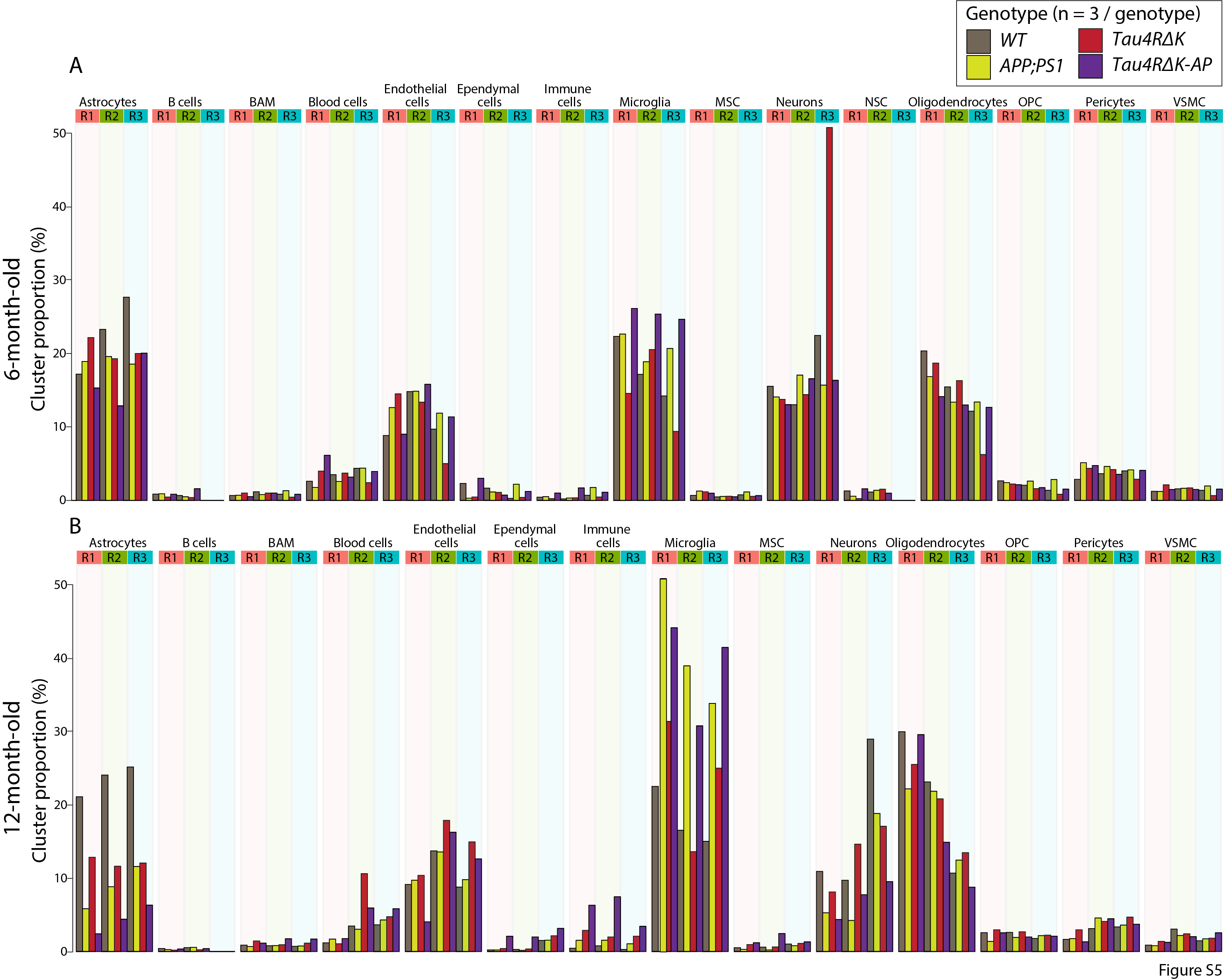

Supplement: Supplementary file 5 — Additional file 5: Figure S5. Distribution of clusters across genotypes. A, Distribution of cell types across 6-month-old scRNA-Seq triplicates. B, Distribution of cell types across 12-month-old scRNA-Seq triplicates. BAM = brain-associated macrophages, MSC = muscle stem cells, OPC = oligodendrocyte precursor cells, VSMC = vascular smooth muscle cells. R1 = Biological replicate 1, R2 = Biological replicate 2, R3 = Biological replicate 3. [file 13024_2022_589_MOESM5_ESM.png]

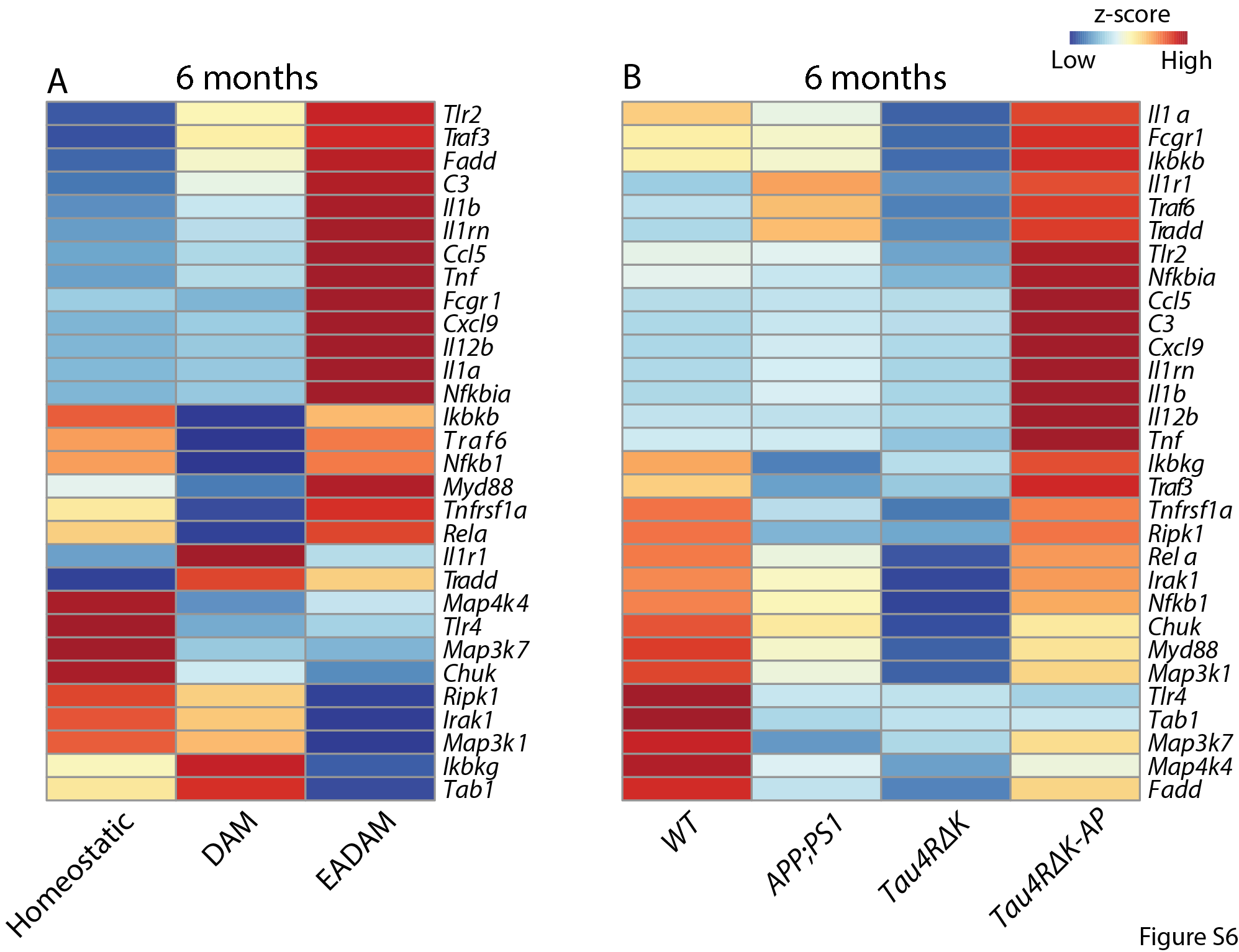

Supplement: Supplementary file 6 — Additional file 6: Figure S6. Analysis of NF-kB pathway genes in 6-month-old AD mouse models. Heatmap plots showing expressions of NF-kB pathway genes at 6-month-old across microglia subtypes (A), and genotypes (B). [file 13024_2022_589_MOESM6_ESM.png]

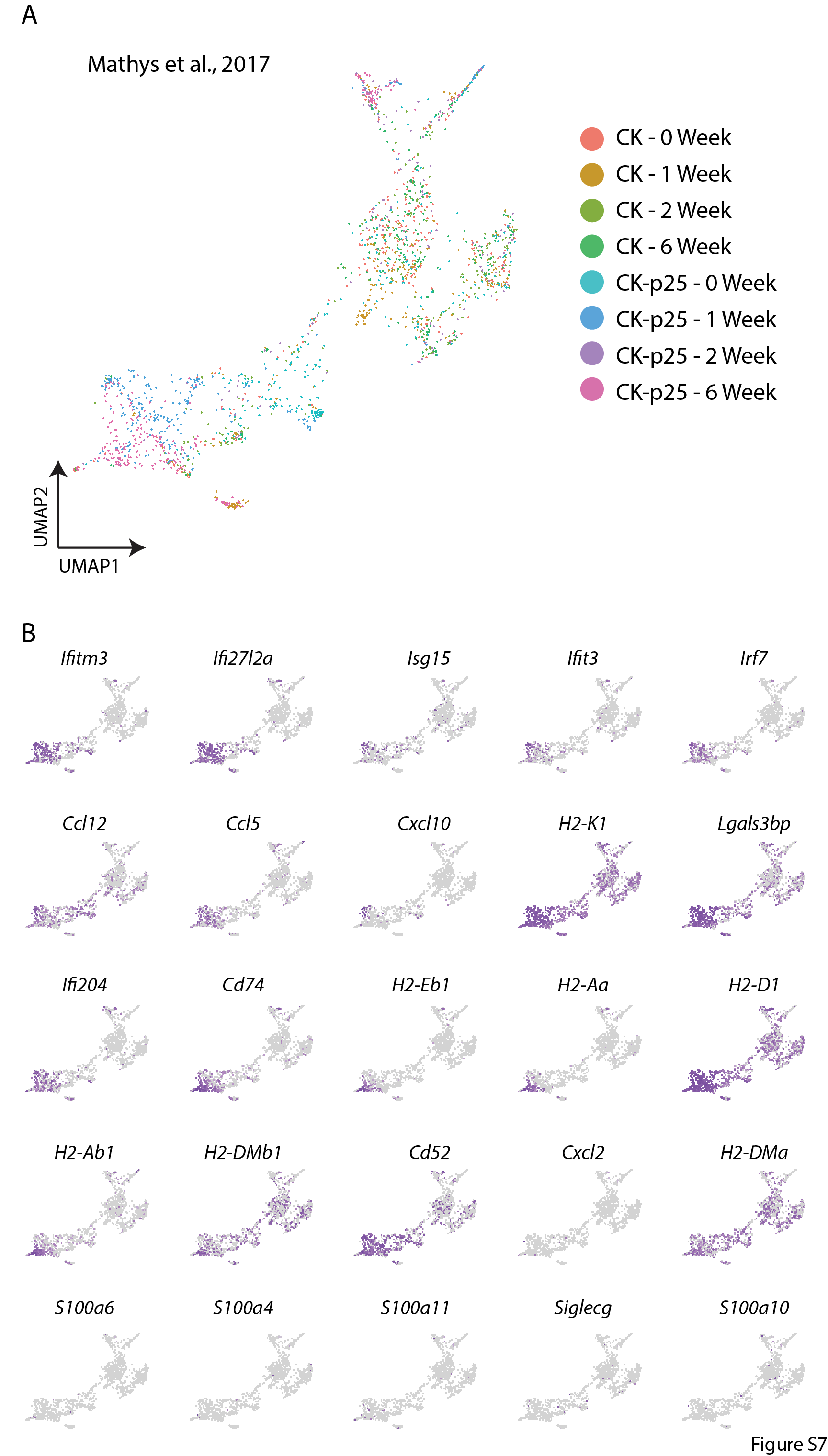

Supplement: Supplementary file 7 — Additional file 7: Figure S7. Analysis of EADAM and LADAM markers in other mouse models of neurodegenerative disease and AD samples. A, UMAP plot showing CK-p25 neurodegenerative scRNA-Seq microglia dataset from Mathys et al., 2017. B, UMAP plots showing EADAM and LADAM-enriched genes. Note that while IFN and MHC genes are present, S100 genes and Siglecg are absent in the dataset. [file 13024_2022_589_MOESM7_ESM.png]

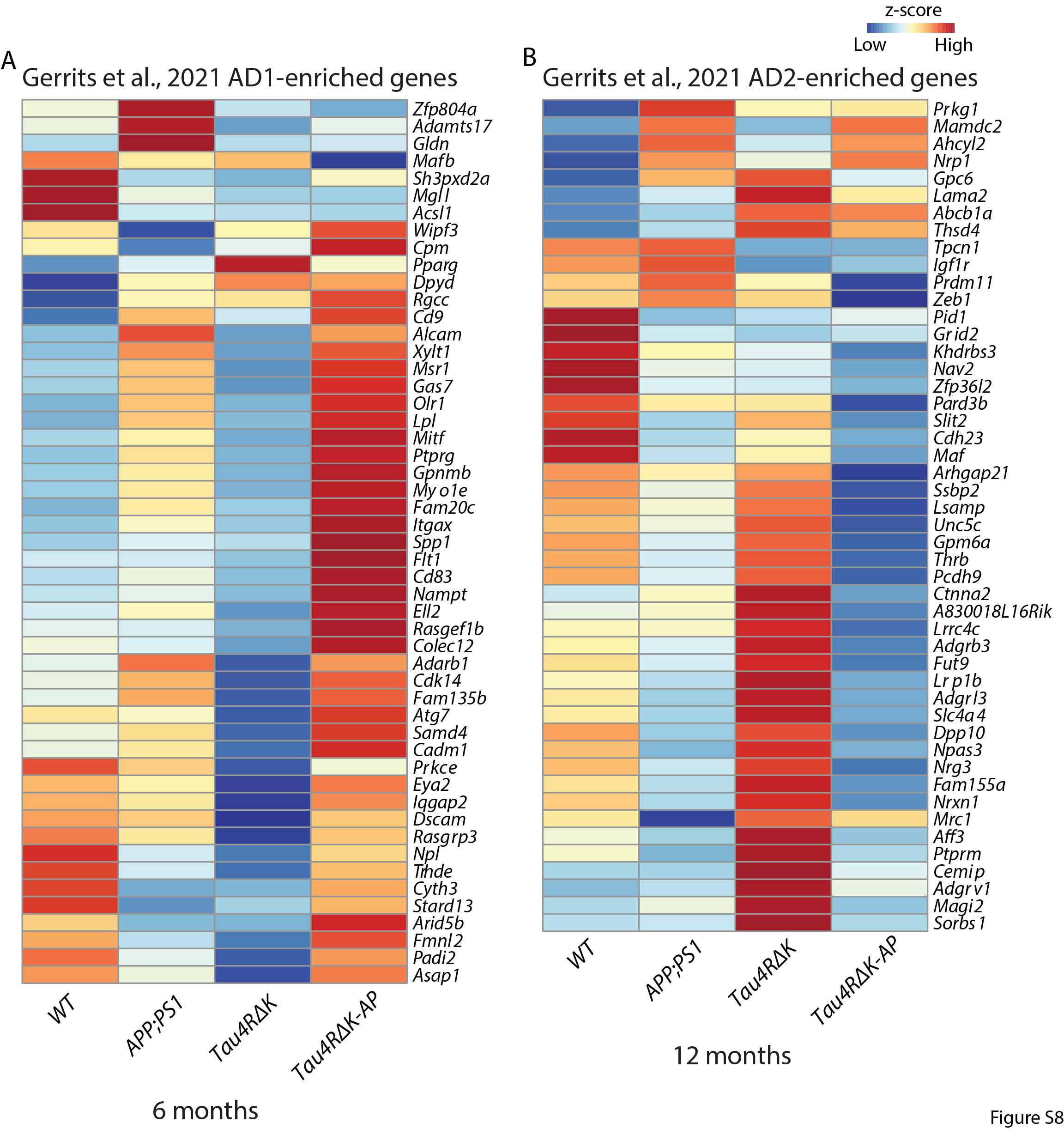

Supplement: Supplementary file 8 — Additional file 8: Figure S8. Analysis of distinct expression profiles that are associated with AD pathology in AD mouse models. Heatmap plots showing homolog expressions of Aβ-associated AD1-enriched (A), and Tau-associated AD2-enriched (B) genes from Gerrits et al., 2021 human snRNA-Seq dataset [59], in 6-month-old 12-month-old, in WT, APP;PS1, Tau4RΔK, Tau4RΔK-AP. [file 13024_2022_589_MOESM8_ESM.png]

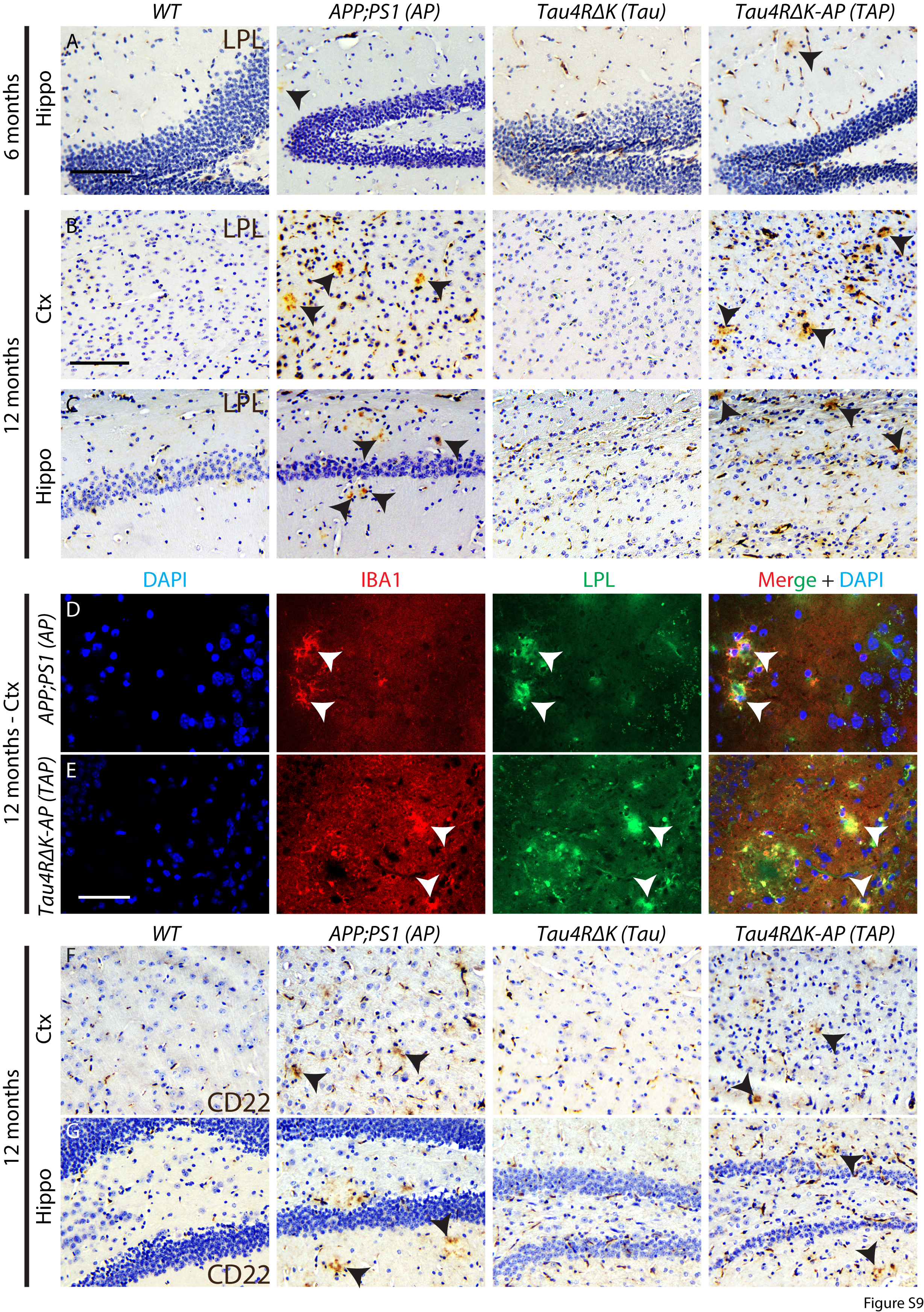

Supplement: Supplementary file 9 — Additional file 9: Figure S9. Histological validation of DAM markers in AD mouse models. A-C, Immunostaining of LPL at 6-month-old (A) and 12-month-old (B, C); in the Cortex (B), and Hippocampus (A, C), in WT, APP;PS1, Tau4RΔK, Tau4RΔK-AP. Black arrowheads indicate examples of LPL-positive cells. D-E, Immunostaining of IBA1 (Red) and LPL (Green) at 6-month-old in the cortex in APP;PS1 (D), Tau4RΔK-AP (E). White arrowheads indicate IBA1 and LPL double-positive cells. F-G, Immunostaining of CD22 (Siglec-2) at 12-month-old; in the cortex (F), and hippocampus (G), in WT, APP;PS1, Tau4RΔK, Tau4RΔK-AP. Black arrowheads indicate examples of LPL-positive cells. Ctx = Cortex, Hippo = Hippocampus. Scale bars = 50 μm. [file 13024_2022_589_MOESM9_ESM.tif]

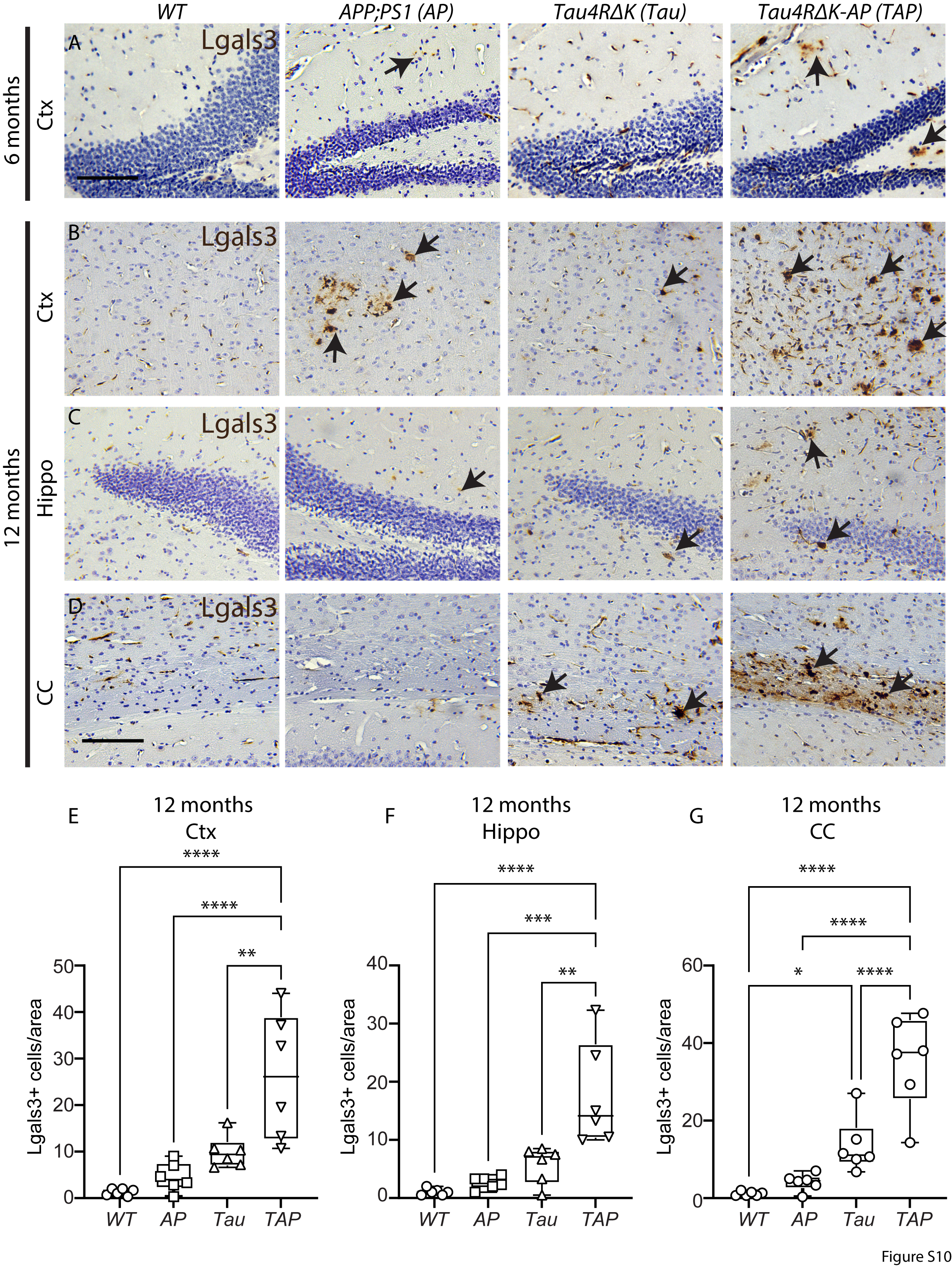

Supplement: Supplementary file 10 — Additional file 10: Figure S10. Histological validation of DAM/LADAM marker in AD mouse models. A-D, Immunostaining of Lgals3 at 6-month-old (A) and 12-month-old (B-D); in the cortex (A, B), hippocampus (C), and corpus callosum (D), in WT, APP;PS1, Tau4RΔK, Tau4RΔK-AP. Black arrowheads indicate examples of Lgals3-positive cells. E-G, Quantification of Lgals3 staining in the cortex (E), hippocampus (F), and corpus callosum (G) at 12-month-old, in WT, APP;PS1, Tau4RΔK, Tau4RΔK-AP. Ctx = Cortex, Hippo = Hippocampus, CC = Corpus Callosum. * P < 0.05, ** P <0.01, *** P < 0.001, **** P < 0.0001. [file 13024_2022_589_MOESM10_ESM.tif]

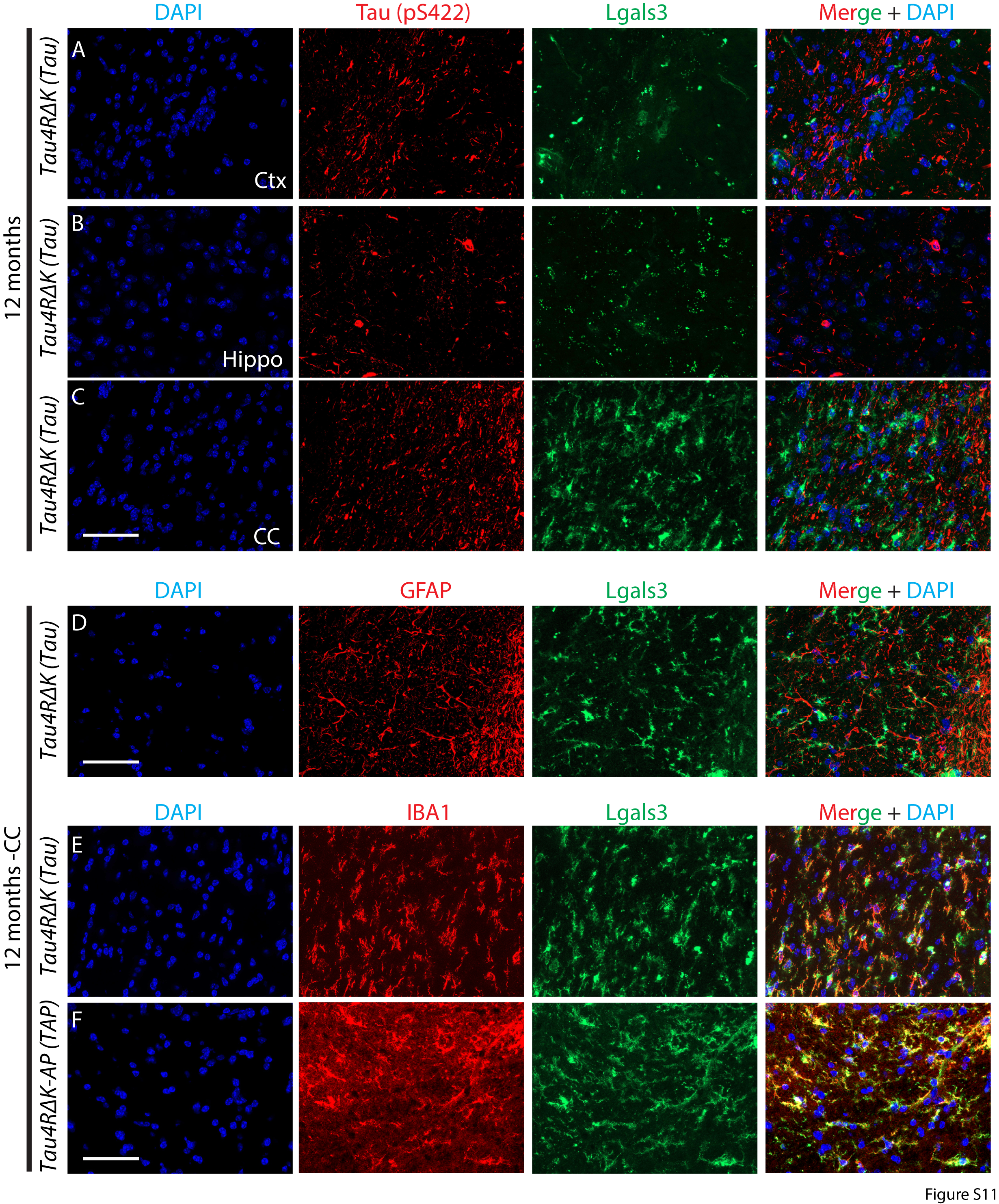

Supplement: Supplementary file 11 — Additional file 11: Figure S11. Histological validation of LADAM marker in AD mouse models. A-C, Immunostaining of Tau(pS422) (Red) and Lgals3 (Green) at 12-month-old in the cortex (A), hippocampus (B), and corpus callosum (C) in Tau4RΔK. D, Immunostaining of GFAP (Red) and Lgals3 (Green) at 12-month-old in the corpus callosum in Tau4RΔK. E-F, Immunostaining of IBA1 (Red) and Lgals3 (Green) at 12-month-old in the corpus callosum in Tau4RΔK (E), Tau4RΔK-AP (F). Ctx = Cortex, Hippo = Hippocampus, CC = Corpus Callosum. Scale bars = 50 μm. [file 13024_2022_589_MOESM11_ESM.tif]

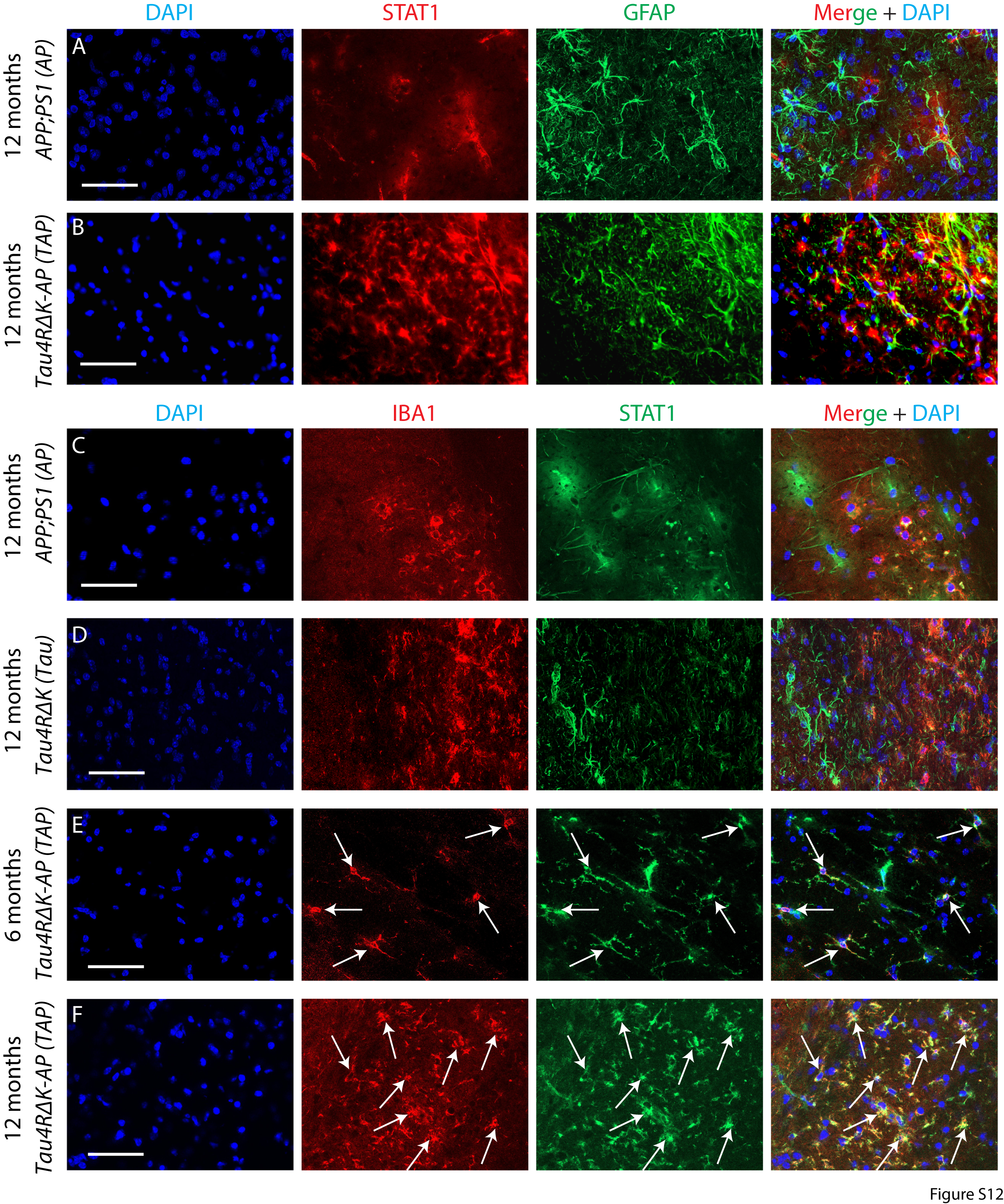

Supplement: Supplementary file 12 — Additional file 12: Figure S12. Histological validation of EADAM/LADAM markers in AD mouse models. A-B, Immunostaining of STAT1 (Red) and GFAP (Green) at 12-month-old in APP;PS1 (A), Tau4RΔK-AP (B). C-F, Immunostaining of IBA1 (Red) and STAT1 (Green) at 6-month-old (E) and 12-month-old (C, D, F) in APP;PS1 (C), Tau4RΔK (D) Tau4RΔK-AP (E, F). Scale bars = 50 μm. [file 13024_2022_589_MOESM12_ESM.tif]

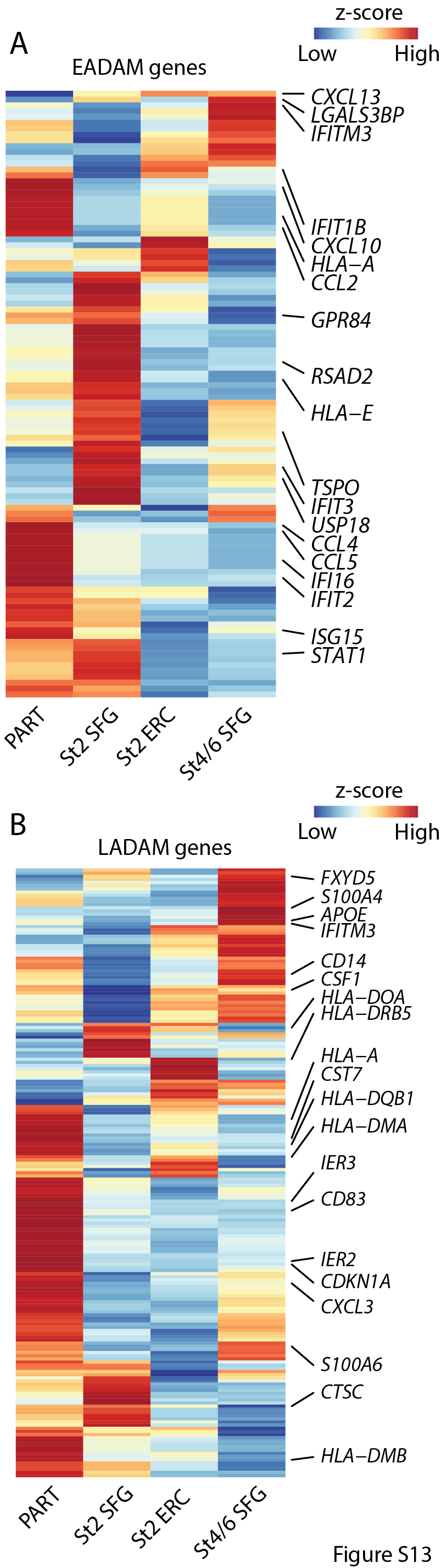

Supplement: Supplementary file 13 — Additional file 13: Figure S13. Analysis of EADAM and LADAM markers in AD samples. Heatmap plots showing homolog expression of EADAM (A), and LADAM (B) genes in the human snRNA-Seq dataset. [file 13024_2022_589_MOESM13_ESM.png]

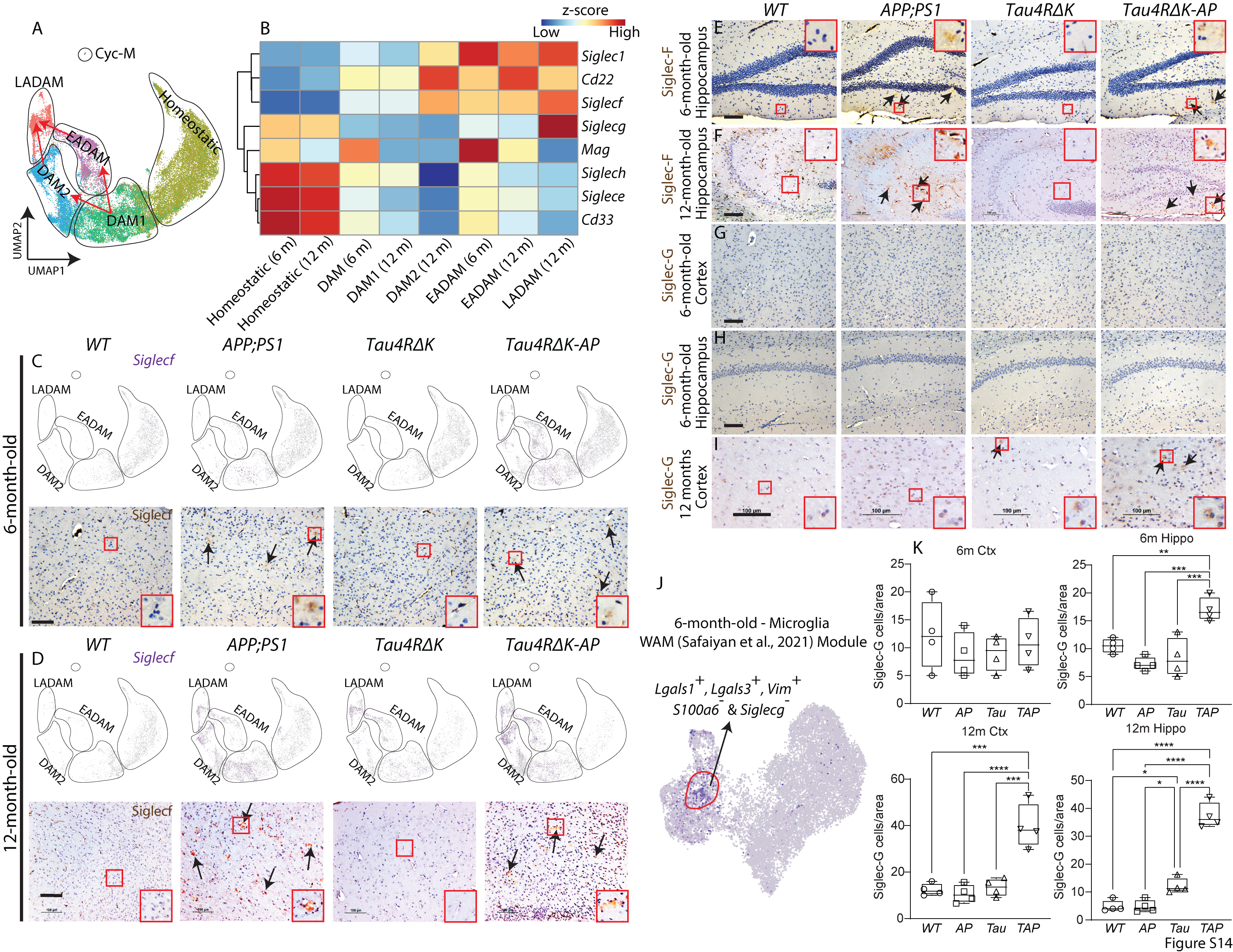

Supplement: Supplementary file 14 — Additional file 14: Figure S14. Siglec-genes show cluster- and genotype-specific expression patterns, and histological validation of Siglec-F and Siglec-G across genotypes in 6 and 12-month-old mice. A, UMAP plot showing microglial clusters in the 6 and 12-month-old cortex (all genotypes). B, Heatmap plot showing Siglec genes expressions across microglial clusters. C, D, UMAP plot showing Siglecf expression in WT, APP;PS1, Tau4RΔK, Tau4RΔK-AP at 6-month-old (C) and 12-month-old (D) (top). Note a higher expression of Siglecf in DAM2 in APP;PS1 and Tau4RΔK-AP mice at 12-month-old. Siglec-F immunostaining was performed in the cortex in WT, Tau4RΔK, APP;PS1, Tau4RΔK-AP at 6-month-old (C) and 12-month-old (D) (bottom). E-I, Immunostaining of Siglec-F (E, F), and Siglec-G (G-I), in WT, APP;PS1, Tau4RΔK, Tau4RΔK-AP; at 6-month-old (E, G) and 12-month-old (F, H, I); hippocampus (E, F, H), and the cortex (F, G, I). J, UMAP plot of 6-month-old microglia, showing a small cluster (highlighted in red) that resembles molecular genes of WAM [25]. K, Quantification of Siglec-G staining in the 6-month-old and 12-month-old cortex and hippocampus. Ctx = Cortex, Hippo = Hippocampus. Red boxes in C, D, E, F, I show high magnification views. Scale bars = 100 μm. * P < 0.05, ** P <0.01, *** P < 0.001, **** P < 0.0001. [file 13024_2022_589_MOESM14_ESM.tif]

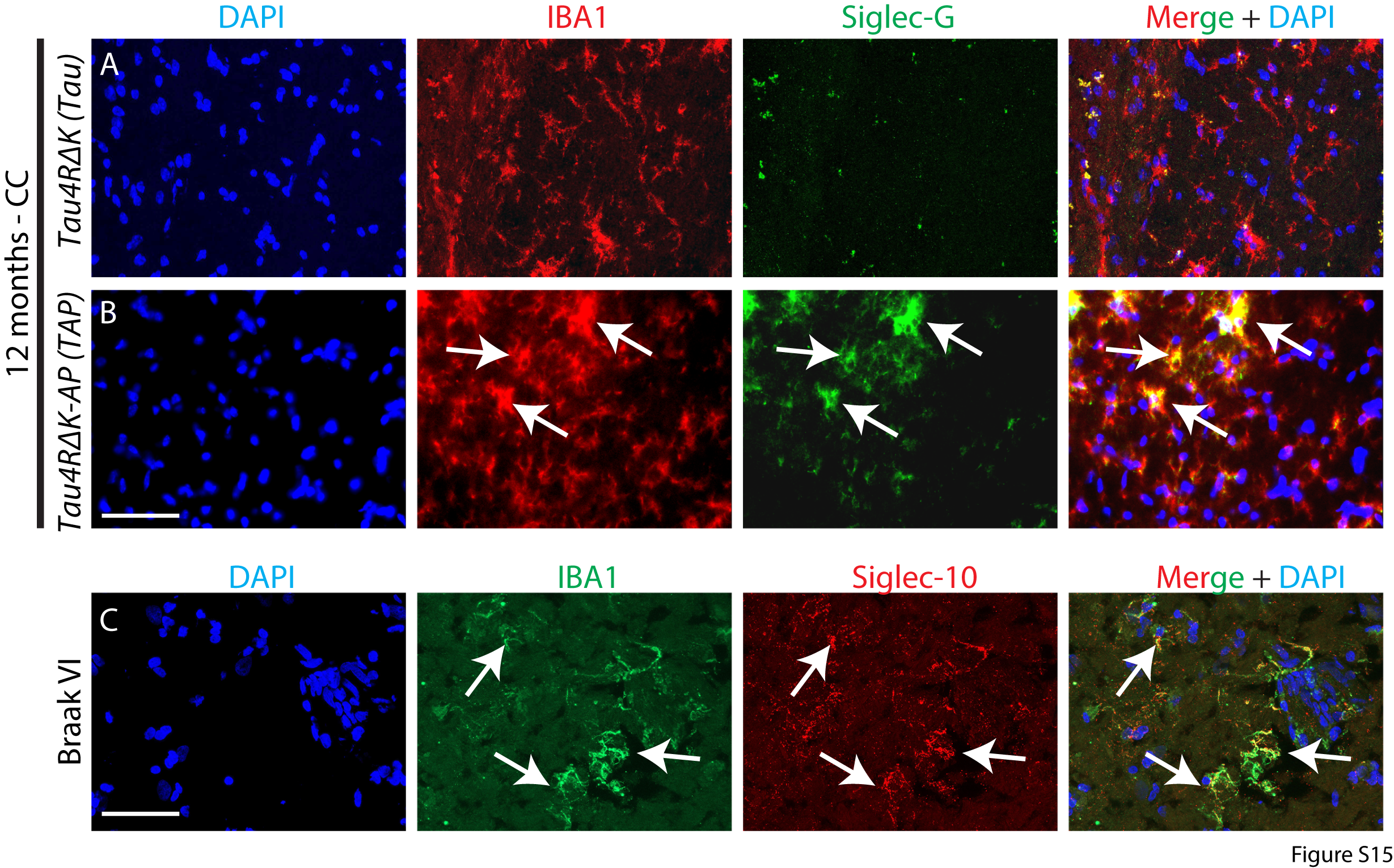

Supplement: Supplementary file 15 — Additional file 15: Figure S15. Histological validation of Siglec-G/Siglec-10 expression in microglia in AD mouse models and human postmortem samples. A-B, Immunostaining of IBA1 (Red) and Siglec-G (Green) at 12-month-old in the CC in Tau4RΔK (A), Tau4RΔK-AP (B). C, Immunostaining of IBA1 (Green) and Siglec-10 (Red) at Braak stage VI. CC = Corpus Callosum. Scale bars = 50 μm. [file 13024_2022_589_MOESM15_ESM.tif]

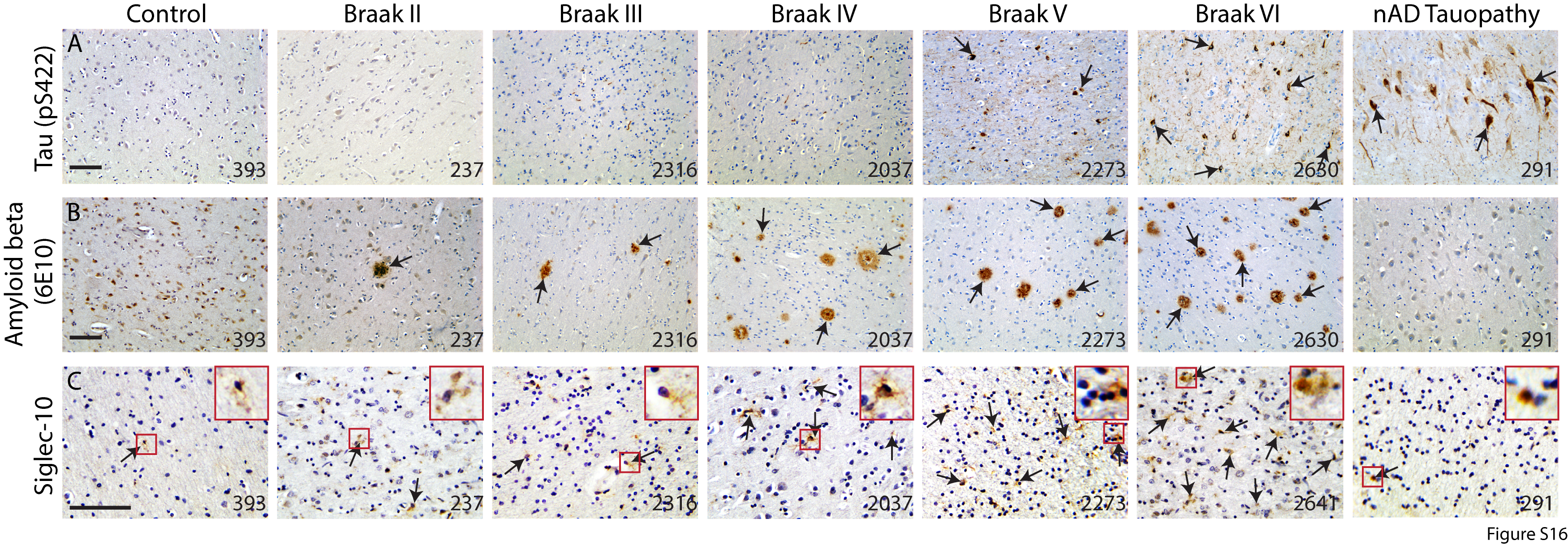

Supplement: Supplementary file 16 — Additional file 16: Figure S16. Histological validation of tau and Aβ pathologies and Siglec-10 during AD progression and in nAD Tauopathy. Immunostaining of Tau (pS422) (A), Aβ (6E10) (B), and Siglec-10 (C) in Control, Braak stage 2, Braak stage 3, Braak stage 4, Braak stage 5, Braak stage 6, and in nAD Tauopathy in the cortex. Numbers indicate BRC# in Table S10. Red boxes show high magnification views. Scale bars = 100 μm. [file 13024_2022_589_MOESM16_ESM.tif]

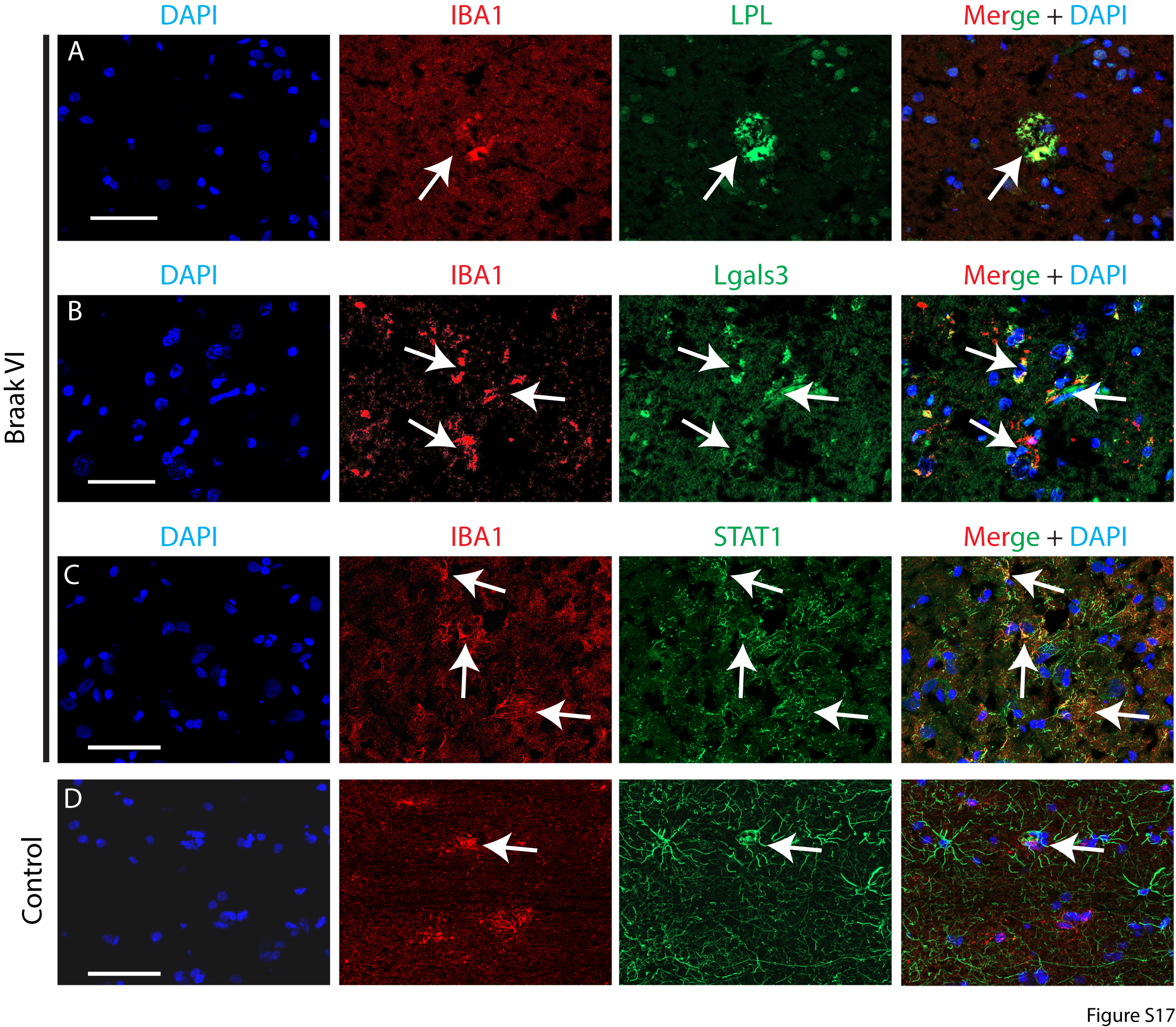

Supplement: Supplementary file 17 — Additional file 17: Figure S17. Histological validation of markers in AD pathology. Immunostaining of IBA1 (Red) and LPL (A, Green); Lgals3 (B, Green); STAT1 (C-D, Green) in Control (D) and AD (A-C). White arrows indicate examples of double-positive cells. Scale bars = 50 μm. [file 13024_2022_589_MOESM17_ESM.tif]
